# Genome-wide in vivo CRISPR screen identifies TGFβ3 as actionable biomarker of palbociclib resistance in triple negative breast cancer

**DOI:** 10.1186/s12943-024-02029-4

**Published:** 2024-06-03

**Authors:** Sophie Poulet, Meiou Dai, Ni Wang, Gang Yan, Julien Boudreault, Girija Daliah, Alan Guillevin, Huong Nguyen, Soaad Galal, Suhad Ali, Jean-Jacques Lebrun

**Affiliations:** Department of Medicine, Cancer Research Program, McGill University Health Centre, Montreal, QC Canada

## Abstract

Triple negative breast cancer (TNBC) remains exceptionally challenging to treat. While CDK4/6 inhibitors have revolutionized HR + breast cancer therapy, there is limited understanding of their efficacy in TNBC and meaningful predictors of response and resistance to these drugs remain scarce. We conducted an in vivo genome-wide CRISPR screen using palbociclib as a selection pressure in TNBC. Hits were prioritized using microarray data from a large panel of breast cancer cell lines to identify top palbociclib sensitizers. Our study defines TGFβ3 as an actionable determinant of palbociclib sensitivity that potentiates its anti-tumor effects. Mechanistically, we show that chronic palbociclib exposure depletes p21 levels, contributing to acquired resistance, and that TGFβ3 treatment can overcome this. This study defines TGFβ3 as an actionable biomarker that can be used to improve patient stratification for palbociclib treatment and exploits the synergistic interaction between CDK4/6 and TGFβ3 to propose a new combinatorial treatment for TNBC.

**Supplementary Information:**

The online version contains supplementary material available at 10.1186/s12943-024-02029-4.

## Introduction

In normal tissue, cellular proliferation, cellular growth, stress management and survival are carefully controlled by stringent cell cycle checkpoints and robust DNA repair mechanisms. The complex transformation of a cell from normal to oncogenic is driven by its acquired abilities to sustain proliferation and to circumvent signalling aiming to stop proliferation, causing a deregulation of its cell cycle [1].

Cyclin-dependent kinases (CDKs) and their associated cyclins are evolutionarily conserved, central regulators of the cell cycle. Their activity is initiated by mitogenic signals and is tightly regulated by cyclin-dependent kinase inhibitors and activated cell cycle checkpoints. CDK4 and CDK6 (hereafter referred to as CDK4/6) have been shown to be essential in mediating breast tumor formation [2, 3]. Cyclin D canonically associates with and activates CDK4/6, which mediates the transition from the G1-phase to the S-phase by phosphorylating and inactivating the retinoblastoma protein (Rb). This releases the E2F transcription factor and drives the transcription of genes responsible for the S-phase transition, including cyclin E [4]. Cyclin E, by binding to CDK2, increases its activity and results in Rb hyperphosphorylation, ultimately driving the cell into S-phase and DNA replication. This process is maintained by endogenous CDK inhibitory proteins of either the INK4 or Cip/Kip family. In breast cancer patients, amplification of the *CCND1* gene may occur in up to 15% of patients, and overexpression of cyclin D1 protein is even more common, occurring in 50% of tumors [5]. For this reason, CDK4/6 has been explored as a potential therapeutic target for breast cancer.

Breast cancer is classified into three major clinical subtypes depending on the expression of the hormone receptors (HR) – estrogen receptor (ER) and progesterone receptor (PR) – and the human epidermal growth factor receptor 2 (HER2). The recent FDA approval of three CDK4/6 inhibitors (CDK4/6is), palbociclib, ribociclib, and abemaciclib, has led to the rapid adoption of targeted treatment of CDK4/6 as first-line or second-line therapy in advanced ER + /HER2- breast cancer. The indication of these inhibitors for ER + /HER2- breast cancer can be attributed to the specific dependency of these tumors on cyclin D1 and CDK4/6 [6]. As is the challenge with many anti-cancer drugs, resistance to CDK4/6 targeted therapies limits their use, ultimately leading to disease spread or relapse. Many studies have been conducted to allow for better clinical decision-making, ranging from identifying the causes of intrinsic resistance, to seeking mechanisms responsible for acquired resistance, and to searching for biomarkers of CDK4/6i efficacy. Patients with triple negative breast cancer (TNBC) have long been ineligible for CDK4/6i therapy because of the absence of ER expression and frequent Rb deletions in TNBC [7]. A phase II clinical trial by DeMichele et al. evaluating palbociclib monotherapy in Rb + metastatic breast cancer found that all four TNBC patients included were refractory to treatment by the study endpoint [8]. Although sample size constraints of the study prevented significant conclusions from being drawn from the TNBC patients tested, the trial results highlight that much remains to be understood about the interplay between TNBC tumor biology and the cell cycle. While independence from CDK4/6 signalling due to Rb deficiency is often linked to TNBCs’ resistance to CDK4/6is, only approximately 35% of TNBCs are Rb-deficient. This means that a great majority of these tumors are Rb-proficient and are thus potential candidates for CDK4/6i therapy [9]. Concordantly, we and others have shown that CDK4/6 inhibition by palbociclib reduces tumor growth in vivo in multiple Rb + TNBC models [10–12]. These findings indicate that there is an avenue worth exploring for CDK4/6i therapy in TNBC; however, there is an unmet need for better biomarkers of response to CDK4/6is. Such predictive markers of drug effectiveness would allow for the identification of a new subset of patients with TNBC who would likely benefit from treatment with CDK4/6is.

This study sought to identify and characterize predictive markers of sensitivity and resistance to palbociclib in TNBC, and to select actionable targets for improving palbociclib efficacy in both TNBC and the general context of breast cancer, through a combinatorial approach. Here, we conducted an in vivo genome-wide CRISPR loss-of-function screen in TNBC to identify genes that could sensitize cells to palbociclib treatment. The enriched gene set (205 genes) was then cross-referenced with microarray data from 38 breast cancer cell lines ranked based on their sensitivity/resistance levels to palbociclib and allowed us to ensure that the gene set is relevant to the broader context of breast cancer, and not limited only to the TNBC subtype. This is important considering the actual clinical context in which the drug is administered.

We aimed to validate the top candidates in vivo using preclinical xenograft models of Rb + TNBC, to confirm the corresponding genes as potential palbociclib sensitizers. We then showed that our top-ranking candidate gene, *TGFB3*, could synergize with palbociclib to generate strong anti-tumor effects both in vitro and in vivo. This synergy is largely achieved through a p21-dependent mechanism, whereby the addition of TGFβ3 induces p21 expression, which further contributes to inhibiting still-active CDK4/6/cyclin D1 and CDK2/cyclin E1 complexes. To further translate our findings to the clinic, we also showed that recombinant human TGFβ3, comparable to avotermin, which has been used in several phase I and II clinical trials for the prophylactic treatment of tissue scarring of the skin, efficiently increased breast tumor response to palbociclib treatments in preclinical models of TNBC.

This study underscores the ability of TGFβ3 levels to predict sensitivity to palbociclib and highlights TGFβ3 as an actionable biomarker capable of improving palbociclib efficacy when administered in combination with palbociclib in TNBC. Our findings also highlight the robustness of the in vivo CRISPR screening and prioritization methods used to identify the effectors of palbociclib sensitivity and pave the way for further investigation into combination treatment approaches.

## Results

**Fig. 1:**
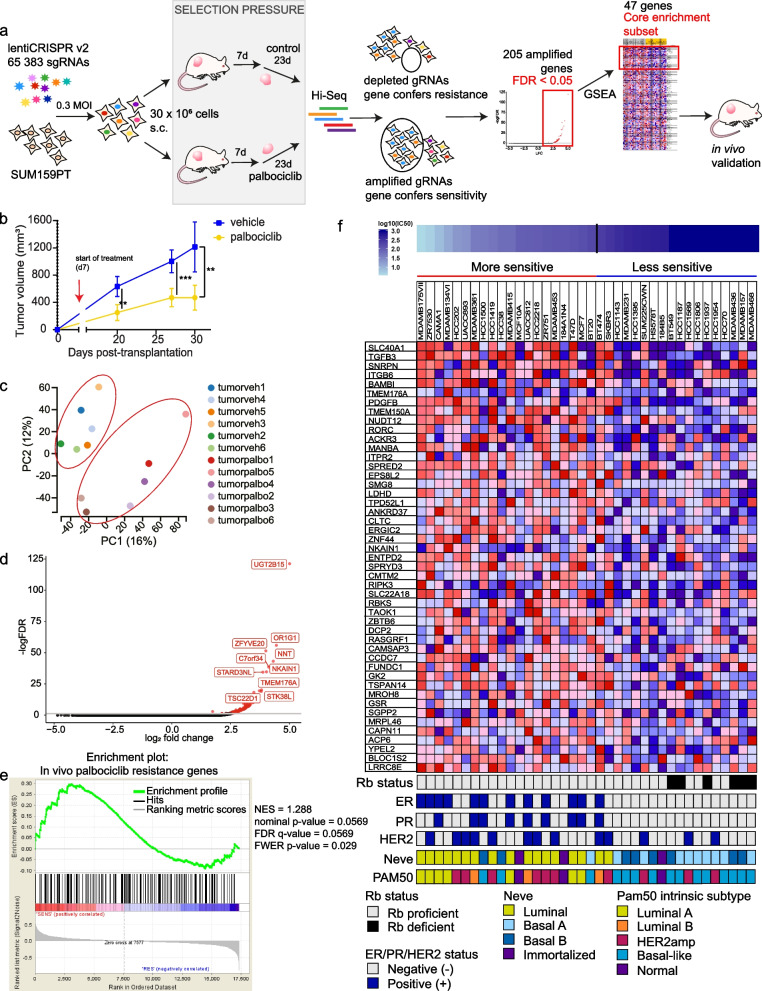
In vivo genome-wide CRISPR knockout screen in TNBC. **a** Schematic representation of the approach used for gene discovery and validation. **b** Average tumor volume in NSG mice measured over 30 days. Intraperitoneal (i.p.) injections of either vehicle or palbociclib started on day 7 post-cell implantation, and lasted 23 days. Mean of three independent infection replicate experiments (*n* = 6, 2 mice per biological replicate). Data are represented as mean ± standard deviation (SD). Significance was calculated using two-sided, unpaired t-test, *p*-value * < 0.05, ** < 0.01, *** < 0.001. **c** Principal component analysis (PCA) of the sgRNAs from the library sequenced in vehicle-treated tumors (*n* = 6), and palbociclib-treated tumor samples (*n* = 6) at day 30 after normalization. **d** 205 sgRNAs were enriched with log2-fold change (LFC) > 0 at false discovery rate (FDR) < 0.05 in palbociclib-treated tumors during the screen. Genes representing significant hits are highlighted in red. **e** Palbociclib sensitivity data was used to rank 38 breast cancer cell lines of varying subtypes, generating two profiles of cell lines, ‘sensitive’ and ‘resistant’. GSEA was used to determine whether 205 sgRNA gene set was significantly enriched in either group of cell lines. Enrichment plot provides the distribution of the enrichment score (green line) of the 205-gene set in the ranked cell lines (sensitive to resistant, left to right). The final, positive normalized enrichment score (NES) at 1.288 indicates significant enrichment of the 205-gene set at FDR < 0.25 in palbociclib ‘sensitive’ cell lines (FDR = 0.0568, *p*-value = 0.0568). **f** Using GSEA, expression levels of the 47 genes (core enrichment subset) are presented here. Cell lines are annotated with clinical information

We aimed to identify clinically relevant genes that mediate palbociclib sensitivity by using an in vivo genome-scale CRISPR/Cas9 loss-of-function screen in a preclinical model of TNBC. We used an Rb-proficient human SUM159PT TNBC cell line [13]. We selected SUM159PT because it is (i) a well-established tumorigenic and metastatic model in vivo (ii) Rb + [14] and thus intrinsically sensitive to CDK4/6 inhibitor treatment, and (iii) representative of TNBC as it harbors PIK3CA and TP53 mutations, two of the most frequently observed mutations in TNBC [15, 16]. As illustrated in Fig. 1a, SUM159PT cancer cells were transduced with the lentiviral pooled genome-scale CRISPR/Cas9 knockout (KO) GeCKOv2 library. GeCKOv2 covers the whole genome with three single guide RNAs (sgRNAs) for each of the 19,050 target genes and 1000 non-targeting control sgRNAs [17, 18]. A low multiplicity of infection (MOI ~ 0.3) was chosen to ensure the integration of only one sgRNA per cell. Due to the sheer number of cells to be transduced, and the complexity of delivering perturbation reagents directly in the host organs of a large number of mice that would have been required to perform a direct in vivo screen, an indirect screen was chosen. Stable knockout cells were thus injected subcutaneously (s.c.) into severely immunodeficient NOD scid gamma (NSG) mice at approximately 400-fold library coverage for each animal in each of the three independent experiments. Tumors were allowed to grow for seven days, until palpable. Mice were then randomized and subjected to intraperitoneal injections of either vehicle or 30 mg/kg palbociclib once daily for five days/week for 23 days. Tumor volume was monitored over the entire 30-day duration of the experiment. Exposure of GeCKO-derived tumors to palbociclib effectively reduced tumor size, illustrating the potency of palbociclib when administered in the in vivo TNBC setting (Fig. 1b). The cell representation samples were sequenced on the day during which the cells were transplanted subcutaneously in mice, to examine the evenness of the library representation. The cell population at day 0 harbored a 99% library representation, indicative of an excellent library coverage (data not shown). Sequencing of tumors revealed a high degree of reproducibility, as demonstrated by the close grouping of principal component analysis (PCA) (Fig. 1c) in six same-condition in vivo biological replicates. PCA again highlighted the relative separation of sgRNA distribution between the untreated and palbociclib-treated samples (Fig. 1c). sgRNAs that were enriched or depleted after in vivo screening under palbociclib selection pressure were then identified. Enriched sgRNAs in palbociclib-treated tumors define genes conferring sensitivity to palbociclib, where loss-of-function mutations in these genes increase overall cell resistance to drug treatment and would thus present novel markers predictive of the palbociclib response. While we did not obtain any significantly depleted sgRNAs, a total of 205 candidate sgRNAs were positively enriched in the palbociclib-treated tumors (Fig. 1d). The sgRNA enrichment profile was generated by filtering sgRNAs with false discovery rate (FDR) < 0.05. Any sgRNAs with fewer than 10 control reads were dropped from the analysis to ensure screen quality and reduce the potential for false positive hits. Gene ontology pathway enrichment analysis performed on the 205 gene list revealed no significantly enriched gene sets or pathways.

To shortlist candidate genes that could best predict palbociclib sensitivity in TNBC, we next cross-referenced our CRISPR screen gene dataset with microarray data from a panel of 38 breast cancer cell lines with varying sensitivities to palbociclib [19]. Cell lines were ranked from most to least sensitive based on palbociclib IC50 values determined in Finn and colleagues' study [7] and correspondingly divided into two groups: ‘more sensitive’ and ‘less sensitive’ to palbociclib. Using Gene Set Enrichment Analysis (GSEA), we sought to determine if the gene set obtained by our screen was enriched in the ‘more sensitive’ cell lines sorted by sensitivity to palbociclib (IC50) [20]. As expected, our 205-gene set was significantly upregulated at FDR < 0.25 in cell lines which are sensitive to palbociclib (FDR = 0.0568) (Fig. 1e). The ‘more sensitive’ cell lines expressed higher levels of genes in our gene set, underscoring the power of our screen to identify genes predictive of palbociclib efficacy across a broad landscape of breast cancer subtypes (Suppl. Figure 1a). Of this gene set, 47 genes formed the ‘core enrichment subset’ as defined by GSEA; genes which contributed most to the positive normalized enrichment score (NES) generated for the entire gene set [20, 21].

We hypothesized that this subset would therefore have the strongest association with palbociclib effectiveness and could serve as a predictive gene signature for palbociclib sensitivity and overall clinical outcomes in patients. We associated the 38 cell lines used in the GSEA with corresponding clinical information. As expected, this cell line ranking coincided with clustering of cell lines based on Rb proficiency, hormone receptor (HR)/HER2 status, and molecular subtype classification, such that known CDK4/6 sensitivity phenotype criteria were fulfilled (Fig. 1f) [22–24]. Indeed, Rb-deficient cell lines clustered together in the ‘less sensitive’ subgroup, as did most cell lines representing the basal subtype of breast cancer. Conversely, HR + and HER2 + cell lines, and cell lines of luminal or HER2 molecular subtype, largely clustered in the ‘more sensitive’ subgroup (Fig. 1f). These findings contributed to our confidence in the screening and the prioritization methods used as they allowed us to situate our results in the context of what is already known. Nonetheless, these results also help strengthen our rationale for the study, showing that palbociclib sensitivity is not simply dictated by ER status or Rb mutation status during patient stratification. We next sought to evaluate whether the 47-gene core enrichment subset could serve as a predictive gene signature for palbociclib sensitivity and overall clinical outcomes in publicly available data sets. We evaluated these genes’ expression patterns in a cohort of patients with breast invasive carcinoma (METABRIC) using cBioPortal [25–27]. We observed a trend towards a decrease in gene expression in the HR-/HER2- (TNBC) subgroup, as compared to the other groups classified by their expression of HR and HER2, although this was not significant (Suppl. Figure 1b). A seemingly lower expression of the 47-gene signature was also observed in the more aggressive basal and claudin-low groups of patient samples, and tended to correlate with higher tumor grade, although this was not significant (Suppl. Figure 1c, d). Taken together, the significant upregulation of the 205-gene set obtained from our in vivo CRISPR/Cas9 screening in the 20 ‘more sensitive’ cell lines underscores the power of the screen to reliably and robustly identify markers of drug effectiveness. These findings strengthen the predictive power of the gene signature defined using our prioritization method, showing that overall lower expression of genes here correlates with poorer clinical outcomes in general, while also promoting palbociclib resistance.

**Fig. 2:**
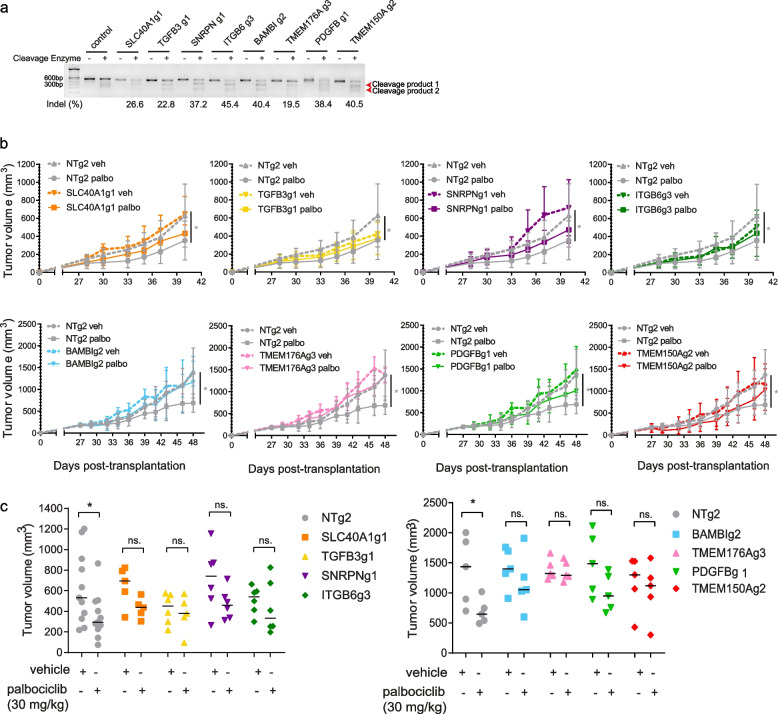
In vivo validation of top candidate genes. **a** Gene modification detection of individual CRISPR-mediated knockouts of top candidate genes. **b** Cells transduced with non-targeting (NT) control or top candidate gene (*SLC40A1, TGFB3, SNRPN, ITGB6, BAMBI, TMEM176A* or *PDGFB, TMEM150A*) KO constructs were transplanted orthotopically into the mammary fat pads of NSG mice. Tumors were palpable before mice from each NT (*n* = 10–22) or targeting group (*n* = 10–12) were randomized into treatment groups (vehicle, *n* = 5–11; palbociclib (30 mg/kg), *n* = 5–11). Mean ± SD tumor volume is shown. Significance was calculated using two-sided, unpaired t-test, *p*-value ns. = nonsignificant, * < 0.05. **c** Tumor volumes of individual mice in each group, NT or targeting a candidate gene, either treated with vehicle or palbociclib at experiment endpoint (*n* = 5). Midlines indicate median tumor volume. Significance was calculated using two-sided, unpaired t-test, *p*-value * < 0.05

Having evaluated the clinical relevance of the 47-gene signature using patient data, we next assessed these genes' ability to modulate the palbociclib response in vivo, using TNBC xenograft models. For this, the eight top-ranking genes of the 47-gene core enrichment subset (*SLC40A1, TGFB3, SNRPN, ITGB6, BAMBI, TMEM176A, PDGFB* and *TMEM150A*) were selected for validation. Briefly, each gene was individually knocked-out in SUM159PT using CRISPR/Cas9 before being orthotopically transplanted in the mammary fat pad of NSG mice, as previously described [10, 17]. Gene modification efficiency was assessed using a SURVEYOR assay from a bulk population of cells, confirming the indel mutations for each KO (Fig. 2a). Once tumors became palpable, daily intraperitoneal injections of the vehicle or 30 mg/kg palbociclib were each administered to five mice within each group, where each group consisted of 10–12 mice per gene knockout. As expected, tumor growth in non-targeting (NT) control mice groups was significantly inhibited by palbociclib by study endpoint (Fig. 2b, c). We found that individual knockout of our target genes effectively made cells more resistant to palbociclib over time (Fig. 2b). By study endpoint, all eight of the eight individual KOs (SLC40A1g1, TGFB3g1, ITGB6g3, BAMBIg2, TMEM176Ag3, PDGFBg1 and TMEM150Ag2) significantly inhibited the palbociclib anti-tumor effect in vivo, defining these genes as key regulators of TNBC response to palbociclib (Fig. 2c).

Having found that the depletion of our top targets generated resistance to palbociclib, we further explored the clinical translatability of our genes to predict the sensitivity of mammary tumors to CDK4/6 inhibitors. Accordingly, we used patient data from the NeoPalAna clinical trial, a single-arm phase II clinical trial evaluating the neoadjuvant use of palbociclib, with an anastrozole backbone, in clinical stage 2 or 3 ER + primary breast cancer [28]. Upon starting the trial, eligible patients received the aromatase inhibitor anastrozole (1 mg daily) for 28 days (Cycle 0). Palbociclib (125 mg daily on days 1–21, Cycle 1) was then added to the treatment regimen on day 1 of cycle 1 (C1D1). Tumor biopsies were collected on C1D1 and 14 days after the start of palbociclib treatment (C1D15). Although all patients were ER + , the only clinical subtype of breast cancer assumed to be responsive to palbociclib, the response to treatment varied in these patients. This illustrates the inadequacy of relying solely on the predictive power of ER positivity. We therefore posited that varying the expression levels of other genes, such as genes from our shortlist, might better predict these varying responses to palbociclib. Gene expression data from total RNA were generated using an Agilent microarray platform during the trial. Here, we compared data from palbociclib-sensitive patients with data from patients deemed palbociclib-resistant at C1D15 because of an inability to achieve complete cell cycle arrest (Ki67 > 2.7%). At C1D1, analysis of gene expression levels revealed lower levels of *SLC40A1* and *TGFB3* in resistant versus sensitive patients (Suppl Fig. 2a). This trend of lower *SLC40A1* and *TGFB3* expression in resistant versus sensitive patients was also observed at C1D15. Some of the remaining genes showed similar trends at both time points, but the overall statistical analysis was difficult to perform given that there were too few patients for whom we had gene expression data in the ‘palbociclib-resistant’ group. These data should therefore be interpreted with caution. Nonetheless, we propose that the trends observed in the expression of the top two genes, *SLC40A1* and *TGFB3*, hint at the potential clinical relevance of our CRISPR screening results in Rb-proficient TNBC in patients with varying Rb statuses in ER + patients.

Analysis of publicly available clinical data on KM Plotter revealed that many of these genes were also correlated with relapse-free survival (RFS) across all breast cancer subtypes [29]. Lower gene expression of *SLC40A1, TGFB3, SNRPN, TMEM176A* and *TMEM150A* was significantly correlated (*p* < 0.05) with lower RFS (Suppl. Figure 2b). This may suggest that lower expression of these genes not only affects the response to palbociclib treatment but is also indicative of a worse overall prognosis for breast cancer patients.

Altogether, these results highlight the robustness of both the prioritization and the screening design used in our study. Furthermore, our in vivo findings may attest to the translatability of these results towards clinical applications, as we found that patients who were resistant to palbociclib did have lower median expression of *SLC40A1* and *TGFB3* in the NeoPalAna trial.

**Fig. 3:**
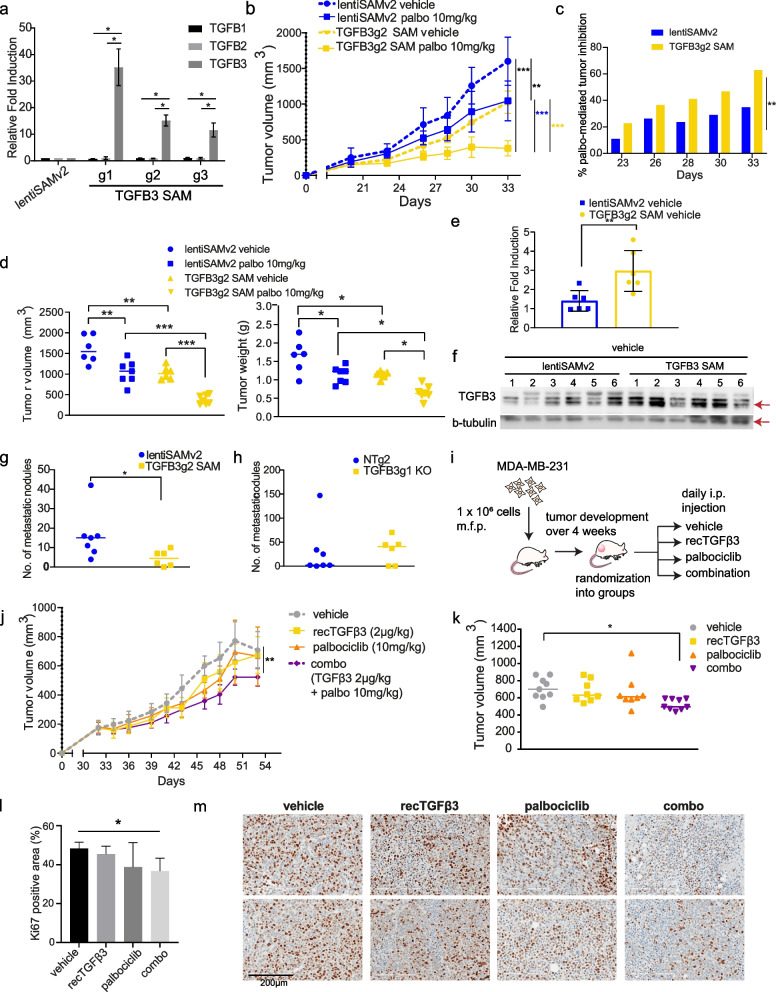
TGFβ3 potentiates palbociclib anti-tumor effect in vivo. **a** mRNA expression levels of *TGFB1, TGFB2* and *TGFB3* in SUM159PT following *TGFB3*-specific overexpression using CRISPR activation (CRISPR/dCas9 SAM) (*n* = 3). Data are represented as mean ± standard deviation (SD). Significance was calculated using two-sided, unpaired t-test, *p*-value * < 0.05. **b** Mice from control (lentiSAMv2) or *TGFB3*-overexpressing (TGFB3g2 SAM) groups (*n* = 13) were each randomized into treatment groups (vehicle, *n* = 6; palbociclib, *n* = 7). I.p. injections of the vehicle treatment or a low dose of palbociclib (10 mg/kg) were administered until study endpoint. Data are represented as mean ± SD. **c** Reduction in tumor growth presented for each group treated with palbociclib, lentiSAMv2 or TGFB3g2 SAM, as compared to the same groups treated with the vehicle. Data are represented as mean, at each timepoint. **d** *left* Tumor volumes of individual mice in each group at study endpoint. *right* Tumor weights of individual mice in each group at study endpoint. Midlines at median. Significance was calculated using ordinary, one-way ANOVA with Tukey’s multiple comparisons test, *p*-value * < 0.05, ** < 0.01, *** < 0.001. **e** Average mRNA expression levels of *TGFB3* in tumors derived from the vehicle-treated control mice (*n* = 6) and the *TGFB3*-overexpressing mice (*n* = 6). Data are represented as mean ± SD. Significance was calculated using two-sided, unpaired t-test, *p*-value * < 0.05, ** < 0.01, *** < 0.001. **f** Protein levels of *TGFB3* (60 kDa) in tumors derived from the vehicle-treated control mice (*n* = 6) and the *TGFB3*-overexpressing mice (*n* = 6). **g** Spontaneous metastasis to the lungs was assessed. Lung nodules were counted and compared in lungs derived from the vehicle-treated control mice (*n* = 7) and the *TGFB3*-overexpressing mice (*n* = 6). Data represent metastatic nodule count per pair of lungs per mouse. Midlines at median. Significance was calculated using nonparametric Mann–Whitney U-test, *p*-value * < 0.05, ** < 0.01, *** < 0.001. **h** The effect of *TGFB3* CRISPR-mediated knockout on lung colonization was assessed. Data represent metastatic nodule count per pair of lungs per mouse. Midlines at median. **i** Schematic representation of the use of recTGFβ3 in combination with palbociclib. MDA-MB-231 TNBC cells were transplanted into the mammary fat pads of NSG mice. Tumors were palpable before mice were randomized into treatment groups: vehicle, *n* = 9; recTGFβ3, *n* = 8; palbociclib, *n* = 8, combo (recTGFβ3 + palbociclib), *n* = 9. **j** Average tumor volume was measured over time. Data are represented as mean ± SD. **k** Tumor volumes of individual mice in each group at study endpoint. Midlines at median. Significance was calculated using ordinary, one-way ANOVA with Tukey’s multiple comparisons test, *p*-value * < 0.05. **l** Quantification of Ki67-positive cells stained by immunohistochemistry in tumor tissues from all four groups. Data are represented as mean ± SD (*n* = 3–4). Significance was calculated using two-sided, unpaired t-test, *p*-value * < 0.05. **m** Representative images of Ki67 staining in two tumors per group

The high ranking obtained by *TGFB3* in the prioritization scheme, the strong negation of the palbociclib effect by *TGFB3* knockout in vivo, along with the inverse relationship observed between *TGFB3* expression and palbociclib resistance in patients led us to further explore the potential value of TGFβ3 as a sensitizer to the palbociclib response. We hypothesized that the effect of palbociclib would be potentiated in *TGFB3*-overexpressing tumors, resulting in a greater growth reduction than in control tumors. Therefore, we applied a gain-of-function approach through activation of the *TGFB3* endogenous gene promoter using the CRISPR/dCas9 Synergistic Activation Mediator (SAM) system, as previously described [10, 30]. As shown in Fig. 3a, we strongly induced *TGFB3* gene expression in SUM159PT cells using three different sgRNAs targeting the *TGFB3* gene promoter, without affecting *TGFB1* or *TGFB2* expression. TGFB3g2 SAM-infected SUM159PT cells were transplanted into the mammary fat pads of NSG mice.

Tumors were grown until palpable and treated daily with a relatively low dose of palbociclib (10 mg/kg, i.p.) or vehicle up to 33 days post-implantation. Here, low-dose palbociclib was used to allow for the observation of a potential synergy between treatment and high TGFβ3 levels. As shown in Fig. 3b, low-dose (10 mg/kg) palbociclib treatment significantly reduced tumor growth in the lentiSAMv2 control tumors. A similar level of effect was observed when *TGFB3* expression was induced in untreated cells (TGFB3g2 SAM vehicle). However, of the mice treated with palbociclib, those with *TGFB3*-overexpressing tumors had significantly lower average tumor growth rates than the control mice (Fig. 3b). Statistical significance of the difference in tumor volume was measured at all timepoints and is provided in Suppl. Figure 3a. This is reflected in the mean palbociclib-mediated tumor growth inhibition in each group of mice at every timepoint investigated, where the palbociclib effect on tumor growth inhibition is significantly greater in *TGFB3*-overexpressing tumors as compared to control mice during the entire experiment (Fig. 3c). This is indicative of a potentiation of the palbociclib effect by TGFβ3. At the study endpoint, palbociclib treatment combined with increased *TGFB3* expression greatly reduced tumor volume compared to that in control mice (lentiSAMv2) treated with palbociclib (Fig. 3d, left panel). Tumors were weighed upon resection, and the results shown in Fig. 3d (right panel) indicate that the anti-tumor effects of palbociclib were also greatly enhanced when *TGFB3* was overexpressed. To verify that the enhanced anti-tumor effect observed in the *TGFB3* SAM tumors was attributable to a sustained increase in *TGFB3* levels, *TGFB3* levels were assessed in excised tumors. *TGFB3* SAM tumors exhibited higher levels of *TGFB3* at both the mRNA level and the protein level than the control tumors (Fig. 3e, f). Taken together, these results suggest that an increase in *TGFB3* expression activates a synthetic lethal interaction upon CDK4/6 inhibition, allowing for greater growth inhibition.

Having thus far only evaluated TGFβ3’s contribution to tumor suppression, we wanted to address the other, pro-metastatic arm of the TGFβ family’s dual role in cancer – a concern due to frequent extrapolation of data relating to TGFβ1’s role in promoting breast cancer to TGFβ3 [31]. The role of TGFβ in providing breast cancer cells with metastatic capabilities – such as inducing epithelial-to-mesenchymal transition and priming cells for extravasation, has been well established for TGFβ1 [32, 33]. However, the TGFβ3 ligand specifically has not been well studied. Thus, we evaluated the effect of *TGFB3* overexpression on the spontaneous metastasis of orthotopically transplanted breast cancer cells to the lungs using the CRISPR/dCas9 SAM system described above. Lung nodules were counted after euthanizing the transplanted mice. Mice overexpressing *TGFB3* showed significantly fewer nodules on average than non-targeting control mice (Fig. 3g). In a follow-up experiment, we assessed the effect of *TGFB3* gene silencing on lung colonization. *TGFB3* KO SUM159PT cells were injected into the tail veins of NSG mice, and lung nodules were counted 38 days after cell injection. We observed a trend towards an increased number of nodules in *TGFB3* KO mice compared to non-targeting control mice (Fig. 3h). Taken together, these data suggest that inducing *TGFB3* gene expression does not adversely affect lung metastasis in vivo, while leading to an increased sensitivity of tumors to palbociclib treatment in vivo*.* This highlights a possible therapeutic avenue for the administration of exogenous TGFβ3*.*

Therefore, we exploited the inherent ease of use of TGFβ3 as a potential treatment, being a naturally occurring ligand. Human recombinant TGFβ3 (recTGFβ3) has previously been developed into an intradermal injectable (avotermin) and has been safely used in phase II and III clinical trials for the prevention of scarring [34]. To validate our findings in another TNBC model and thereby broaden the scope of the implications of our findings, we assessed recTGFβ3/palbociclib anti-tumorigenic effects when administered alone or in combination in preformed MDA-MB-231-derived mammary tumors. MDA-MB-231 is a poorly differentiated, aggressive TNBC cell line derived from the pleural effusion of a 51-year-old Caucasian female [35]. These cells were transplanted into the mammary fat pads of NSG mice, which were then randomized into four groups. Either the vehicle, human recTGFβ3 alone (2 µg/kg), palbociclib alone (10 mg/kg), or a combination of recTGFβ3 (2 µg/kg) and palbociclib (10 mg/kg) was administered intraperitoneally to mice in each group (Fig. 3i). Treatment was initiated 33 days after transplantation, once the tumors were palpable and administered daily. The smallest average tumor volume was observed in the combination group (Fig. 3j). By the endpoint, mice from the groups treated with suboptimal doses of either recTGFβ3 alone or palbociclib alone showed comparable tumor volumes to mice in the control group, whereas the recTGFβ3 + palbociclib combination group had significantly smaller tumors than the control group (Fig. 3k, Suppl. Figure 3b). Moreover, analysis of the proliferation index (Ki67) by immunohistochemistry in these tumors revealed that the combination treatment significantly reduced the proportion of proliferating cells as compared to the vehicle (Fig. 3l, m). This is reflective of tumor volume at endpoint, as neither palbociclib alone nor recTGFβ3 alone significantly reduced cell proliferation in vivo, indicating a potential synergy between the two treatments when administered together. These findings highlight the clinical relevance of TGFβ3 as a synthetic lethal target in our screen for its role in potentiating the anti-tumor effects of palbociclib when administered as a recombinant protein. They indicate the ease with which TGFβ3 could be administered in the clinic in combination with palbociclib to achieve significant tumor growth inhibition using low doses of either treatment. This could potentially help avoid unwanted adverse effects of using high individual doses while allowing for on-target inhibition of tumor growth unachievable at low doses of palbociclib.

Having shown that both *TGFB3* overexpression and the use of recTGFβ3 significantly promoted the palbociclib response in reducing tumor growth (Fig. 3), we sought to gain insight into the molecular mechanism by which these two drugs work together. To better understand the nature of the relationship between palbociclib and recTGFβ3, we assessed combinatorial synergy using drug matrix assays in multiple Rb + TNBC cell lines: SUM159PT, SUM229PE, and MDA-MB-231. To start to address this, dose–response analyses with TGFβ3 or palbociclib alone were performed in these TNBC cell lines. As shown in Suppl. Figure 4a, TGFβ3 stimulation of the cells only produced a modest effect that plateaued at approximately 20% growth inhibition. Palbociclib efficiently reduced cell viability within a given concentration range (Suppl. Figure 4b). Ultimately, dose ranges of palbociclib (12.5 nM to 400 nM) and recTGFβ3 (3.13 pM to 100 pM) were used alone or in combination and cell proliferation was assessed by crystal violet staining.

**Fig. 4:**
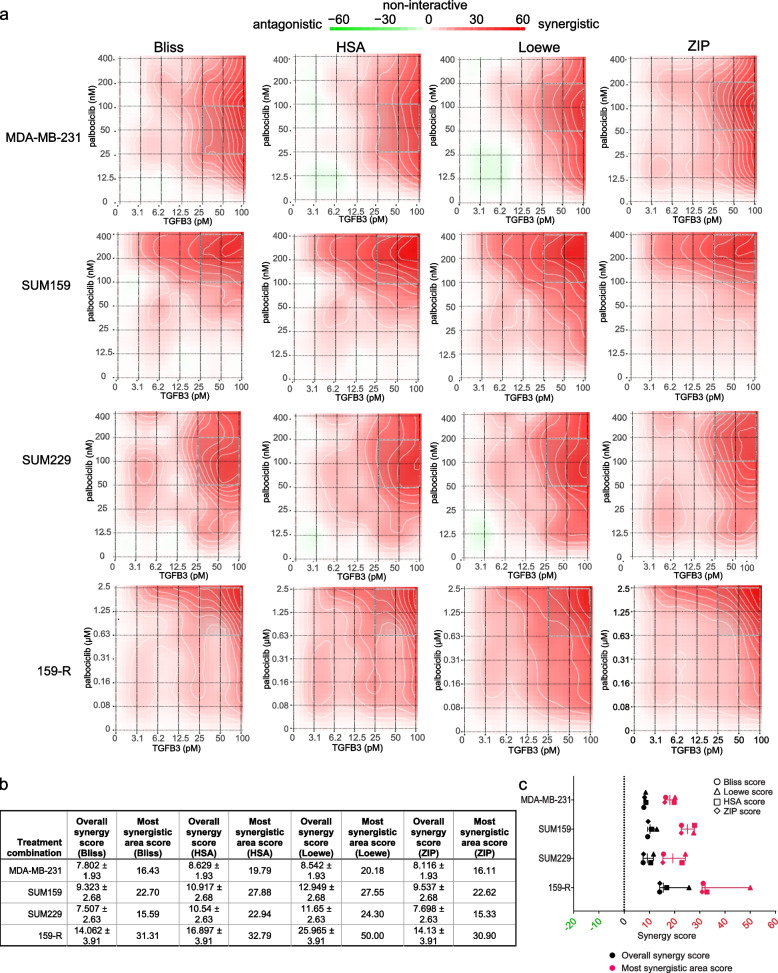
Combination of recombinant TGFβ3 and palbociclib synergistically inhibits TNBC cell proliferation in vitro. **a** Synergy between palbociclib and recTGFβ3 dose combinations was calculated based on four reference models (Bliss, HSA, Loewe, ZIP) using SynergyFinder in four TNBC cell lines (MDA-MB-231, SUM159PT, SUM229PE, 159-R). Synergy maps highlight areas of synergistic (red) or antagonistic (green) interactions between given concentrations of either agent. Grey boxes indicate the area of maximum synergy observed. Mean of a minimum of three independent replicate experiments for each cell line (*n* ≥ 3). **b** ‘Overall synergy scores’ and ‘Most synergistic area scores’ presented for each drug matrix shown in **a**. Data are represented as score ± 95% confidence interval. **c** Dot plots show overall synergy scores (black) or most synergistic area scores (pink) for each cell line, with each dot representing the score obtained using the indicated reference model. Midlines represent median scores. Outer vertical lines correspond to minimum and maximum scores obtained. A zero ‘0’ score indicates no interaction between the two agents

We used four reference synergy models to assess combinatorial effects in our study: Bliss, Highest Single Agent (HSA), Loewe, and Zero Interaction Potency (ZIP). Each of these models uses different formulas and assumptions to calculate drug combination synergy [36]. Interestingly, we found that for all cell lines tested, overall synergy was observed across the dose combinations tested, with scores greater than 10 indicating a strong likelihood of a synergistic relationship [36] (Fig. 4a). Notably, cotreatment attained a level of synergy that could be reproducibly obtained using all four models tested. The highest degrees of synergism tended to occur at the lower concentrations used for palbociclib, as denoted by the grey rectangles in each graph and the ‘Most synergistic area score’ (Fig. 4a, b). The percentages of treatment-induced proliferation inhibition for each pairwise comparison in the drug matrices presented help underscore the impact of the combination treatment in each cell line (Suppl. Figure 4d). This further highlights the clinical relevance of our findings, where submaximal doses of palbociclib could be administered, limiting the associated side effects and reducing the need for treatment cycle delays, along with TGFβ3, to achieve an even greater anti-proliferative effect than palbociclib alone. This is especially relevant in a context where cancer patients are subjected to many treatment-associated toxicities, both with palbociclib and radiotherapy or chemotherapy treatments [37].

We then investigated whether recTGFβ3 could be used to resensitize cells to palbociclib in a model where cells had become resistant to palbociclib due to chronic exposure to the drug. To this end, we first generated a palbociclib-resistant SUM159PT cell line (159-R) by treating SUM159PT with gradually increasing concentrations of palbociclib over four months. A dose–response curve evaluating palbociclib response in 159-R was used to confirm palbociclib resistance (Suppl. Figure 4c). We performed drug matrix assays using palbociclib concentrations ranging from 78 nM to 2.5 µM, while TGFβ3 concentrations ranged from 3.13 pM to 100 pM. Although higher concentrations of palbociclib were necessary in 159-R to generate a similar level of response to the low doses of palbociclib used in parental SUM159PT, we chose to keep the same range of recTGFβ3 concentrations to determine whether resistant cells could be resensitized to palbociclib at the same low concentrations. We found that not only could resistant cells be resensitized to palbociclib by cotreatment with recTGFβ3, but that TGFβ3 could synergize with the effects of palbociclib. Indeed, in 159-R, overall synergy was achieved for the drug concentration ranges tested using all four algorithms (Fig. 4a, b, Suppl. Figure 4d). As demonstrated in Fig. 4c, the robustness of this interaction is made evident by the high synergy scores obtained in all cell lines, regardless of previous exposure to palbociclib, and across all algorithms for the ‘Overall synergy scores’ (black) as well as ‘Most synergistic area scores’ (pink). The potential noninteractive zone (dotted line) was excluded from the range of scores obtained for every synergy score analysis (Fig. 4c). The synergy demonstrated in the treatment-naïve context helps to characterize the interplay observed in the in vivo study, demonstrating that the combination of recTGFβ3 + palbociclib treatment leads to the greatest tumor growth inhibition. Most importantly, this synergy is still achieved when cells are desensitized to palbociclib through chronic exposure to the drug.

**Fig. 5:**
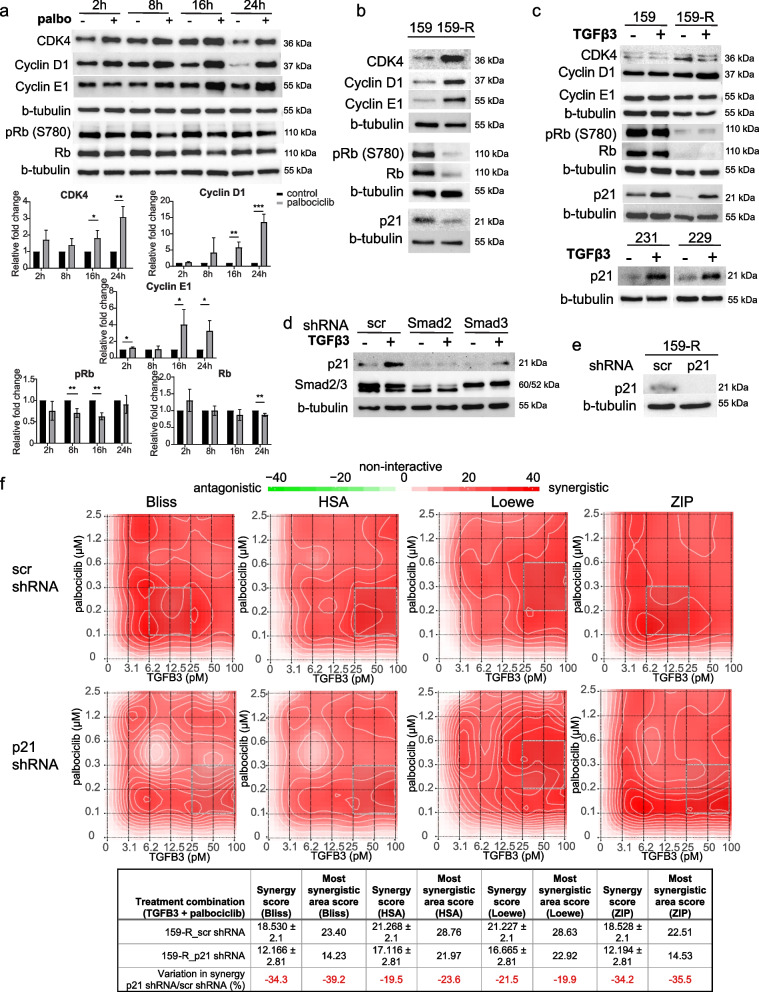
TGFβ3 synergizes with palbociclib in a p21-dependent way*.* **a** SUM159PT cells were treated with palbociclib (100 nM) for 2 h, 8 h, 16 h and 24 h and protein lysates were assessed for known CDK4/6i resistance markers (CDK4, cyclin D1, cyclin E1, Rb, phospho-Rb (S780)) by immunoblotting. Relative fold changes in protein levels, compared to untreated cells at each timepoint, were calculated (*n* = 3). Data are represented as mean ± SD. Significance was calculated using two-sided, unpaired t-test, *p*-value * < 0.05, ** < 0.01, *** < 0.001. **b** SUM159PT (159) and 159-R cells were assessed for known CDK4/6i resistance markers, as well as p21, by immunoblotting. **c** *top* SUM159PT and 159-R cells were treated with recTGFβ3 (100 pM) for 24 h and resulting changes in known CDK4/6i resistance markers and p21 were measured by immunoblotting. *bottom* MDA-MB-231 (231) and SUM229PE (229) cells were treated with recTGFβ3 (200 pM) for 24-48 h and resulting changes in p21 were measured by immunoblotting. **d** SUM159PT cells were transduced with plasmids encoding control (scramble, scr), Smad2-specific, or Smad3-specific short hairpin RNAs (shRNA). Protein levels of p21 and total Smad2/3 were measured by immunoblotting. **e** SUM159PT cells were transduced with plasmids encoding control (scr) and p21-specific shRNA. Protein levels of p21 were measured by immunoblotting. **f** SUM159PT scr shRNA-infected or p21 shRNA-infected cells were treated with varying combinations of palbociclib and recTGFβ3 concentrations. Synergy between dose combinations was calculated using SynergyFinder. *upper* Synergy maps highlight areas of synergistic (red) or antagonistic (green) interactions between given concentrations of either agent. Grey boxes indicate the area of maximum synergy observed between given recTGFβ3 and palbociclib dose combinations. *lower* ‘Overall synergy scores’ and ‘Most synergistic area scores’ presented for each drug matrix shown above. Data are represented as score ± 95% confidence interval (*n* = 3). Percentage variation in synergy score (score obtained in p21 shRNA cells/score obtained in scr shRNA cells) is also shown (red)

To understand the molecular mechanisms underlying the synergism between palbociclib and TGFβ3 growth inhibitory effects in TNBC, we examined the effects of palbociclib on the expression levels of cell cycle regulators. Palbociclib treatment of SUM159PT cells over 24 h led to significant time-dependent increases in established resistance markers, such as CDK4, cyclin D1 and cyclin E1, along with concomitant decreases in Rb and phospho-Rb (Ser780) (Fig. 5a). The various times at which these changes in protein levels occurred may reflect the indirect nature of these changes in protein levels. Of note, observable and significant changes in phosphorylation of Rb occurred earlier in the time course, whereas a significant decrease in Rb levels was observed after 24 h only (Fig. 5a). We observed no consistent changes in CDK6 nor the CDK inhibitor CDKN1B (p27) over 24 h. For CDKN2A (p16) and CDKN1C (p57), we found there was no detectable signal. However, there were changes in protein levels of the other phases of the cell cycle, especially later in the time course (Suppl. Figure 5a). Accordingly, these decreases in CDK1, cyclin A1, cyclin B1, and PLK1 were in line with the decrease in proportion of cells which proceeded to S-phase and continued cycling through the cell cycle after addition of palbociclib (Suppl. Figure 5b). Indeed, following cell cycle analysis by flow cytometry, it is clear that treatment with palbociclib arrests cells in G1, but that the induction of G1 arrest is strongest and significant upon the addition of recTGFβ3, which also entails a significant decrease in the proportion of cells in S-phase (Suppl. Figure 5b).

To next determine whether these changes in cell cycle marker expression would be transposed in the long-term palbociclib acquired resistance context, we compared their levels in naïve and resistant cells that had undergone chronic exposure to the drug, in SUM159PT and 159-R, respectively. As shown in Fig. 5b, strong increases in CDK4, cyclin D1, and cyclin E1, along with a stark decrease in Rb and p-Rb expression, were observed in the resistant cells, indicating that the effects of chronic palbociclib exposure mimicked the changes in marker levels observed in the short-term acquired context. We also found that palbociclib decreased the expression of the cell cycle inhibitor CDKN1A (p21). This defines p21 as a palbociclib target and is consistent with decreased palbociclib efficacy and short-term acquired resistance.

The TGFβ family of ligands acts as potent tumor suppressors notably by inducing CDK inhibitors (CDKIs) [38]. Thus, we examined whether TGFβ3 could modulate the expression of the CDK inhibitor p21 in both parental and palbociclib-resistant SUM159 cells. As shown in Fig. 5c, TGFβ3 strongly induced p21 expression in multiple TNBC cell lines, as demonstrated in SUM159PT, MDA-MB-231 and SUM229PE. Furthermore, it restored p21 levels in palbociclib-resistant cells, suggesting that TGFβ3-mediated p21 expression induction contributes to the synergism observed between palbociclib and recTGFβ3. This is also exhibited at the mRNA level, where treatment with recTGFβ3 significantly induces p21 levels in SUM159PT and, to an even greater extent, in 159-R (Suppl. Figure 5c). At the basal level, without recTGFβ3 treatment, there is a significant decrease in p21 in cells chronically exposed to palbociclib, 159-R, at the mRNA level (Suppl. Figure 5c). This is reflected at the protein level as well (Fig. 5c). Therefore, we further addressed the specific role and contribution of p21 in mediating these effects. First, we determined that the effect of p21 upregulation by TGFβ3 was Smad2/3-dependent. When Smad2 and Smad3 were knocked down individually in SUM159 cells, the TGFβ3-mediated increase in p21 level was diminished (Fig. 5d). Given that Smad2/3 induction of p21 occurs through the well-established canonical Smad signaling pathway shared by all TGFβ isoforms, we asked whether the synergy observed between TGFβ3 and palbociclib could also be observed between TGFβ1 and TGFβ2 with palbociclib. We tested whether these isoforms could confer similar synergistic effects on palbociclib efficacy in MDA-MB-231 and SUM159PT cell lines and found that all three TGFβ isoforms demonstrate a similar effect on palbociclib efficacy (Suppl. Figure 5d). This is in line with the proposed mechanism of action underlying the synergy between TGFβ3 and palbociclib, which occurs through a mechanism common to all three isoforms.

In defining this relationship between Smad2/3 signaling and p21 expression, we examined whether the decrease in p21 observed in palbociclib-treated cells was also mediated through canonical TGFβ Smad signaling. We observed no added contribution to phosphorylation of Smad2/3 or change in total Smad2/3 following palbociclib treatment alone or in combination with recTGFβ3 (Suppl. Figure 5e). Next, we sought to determine whether p21 was at least partially responsible for the synergy observed between palbociclib and TGFβ3 by knocking down p21 in 159-R cells using a p21-specific shRNA (Fig. 5e). Using a drug matrix to characterize the drug-response relationship between a range of pairs of recTGFβ3-palbociclib doses, we found that the synergy scores for the entire matrix tested (‘Overall synergy scores’) strongly decreased with all algorithms – by as much as 34.3% (Bliss) – in the absence of p21 (Fig. 5f). Similarly, all ‘Most synergistic area scores’ in p21 knockdown cells decreased by as much as 39.2% (Bliss) for a given algorithm (Fig. 5f), highlighting the dependence, albeit partial, of TGFβ3-palbociclib synergy on p21.

Altogether, we showed that known cell cycle markers, such as CDK4, cyclin D1 and cyclin E1, are upregulated as early as 2 h following palbociclib treatment, leading to an overall increase in the components necessary for active cyclin/CDK complexes. We also observed a striking decrease in the level of p21 upon chronic exposure to palbociclib, highlighting an additional route by which cells may become desensitized to palbociclib treatment over time. Stimulation of these chronically exposed cells (159-R) with TGFβ3 increased p21 levels and overcame the downregulation of p21 induced by chronic exposure to palbociclib. Finally, we showed that the TGFβ3-mediated increase in p21 is Smad2/3-dependent and plays an important role in the synergism observed between palbociclib and TGFβ3 in TNBC.

**Fig. 6:**
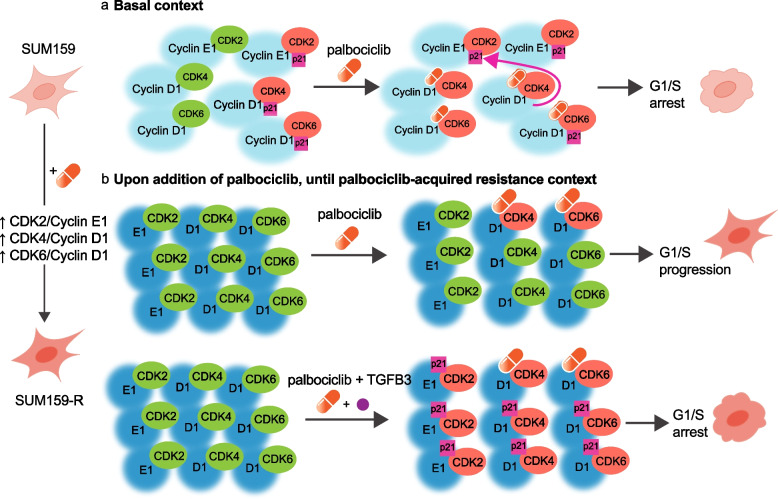
Schematic diagram depicting TGFβ3-palbociclib synergy. **a** In the basal context, cells maintain a balance between active (green) and p21-bound, inactive (red) CDK/cyclin complexes. In the presence of palbociclib (orange capsule), CDK4/6 kinase activity is inactivated, and p21 (pink box) bound to CDK4 is released and preferentially displaced to CDK2. This inactivates CDK2/cyclin E complexes and leads to overall cell cycle arrest. **b** *upper* When cells undergo prolonged exposure to palbociclib, key cell cycle regulators (CDK4, cyclins D and E) are upregulated, while p21 expression is strongly inhibited. Some CDK/cyclin complexes are inactivated (red), but the overall imbalance in active CDK4/cyclinD1 and CDK2/cyclinE1 complexes (green) leads to decreased responsiveness of cells to palbociclib, acquired resistance to the drug, and continued cell cycling. *lower* When TGFβ3 is added in the presence of palbociclib, p21 expression levels are restored through TGFβ3 signalling. The increase in p21 by TGFβ3 synergizes with palbociclib’s mechanism of action, allowing for the inactivation of all remaining active CDK/cyclin complexes (red), and ultimately leading to cell cycle arrest

Based on these findings and previous literature, we propose a mechanistic model for the synergism between TGFβ3 and palbociclib. First, in the basal context, cells maintain a balance between active (green) and p21-bound inactive (red) CDK/cyclin complexes. In the presence of palbociclib, CDK4/6 kinase activity is blocked by the inhibitor, while p21 bound to CDK4 is released and displaced to CDK2, inactivating CDK2/cyclin E complexes, and leading to cell cycle arrest [39] (Fig. 6a). However, upon prolonged exposure to palbociclib, the expression of key cell cycle regulators (CDK4, cyclins D and E) is induced while p21 expression is strongly inhibited, as demonstrated in Fig. 5b. Considering that the increase in the individual expression of key regulators known to bind together, we propose that this implies an increase in the number of complexes formed, and notably, an imbalance in active CDK4/cyclin D1 and CDK2/cyclin E1 complexes (Fig. 6b, upper panel). This progressively leads to acquired palbociclib resistance and reduced drug efficacy. In the presence of both palbociclib and TGFβ3, synergy occurs, where p21 expression levels are restored through TGFβ3, allowing for inactivation of all remaining active CDK/cyclin complexes and thus an increase in p21-bound – thus inactivated – complexes (Fig. 6b, lower panel). This leads to an improved palbociclib response and cell cycle arrest in vitro, ultimately leading to the greater inhibitory effect of the combination treatment observed in vivo*.*

## Discussion

Over the last decade, an increasing amount of evidence supporting a clear clinical benefit of CDK4/6is has led to a rising rate of prescription of these drugs for ER + /HER2- breast cancer. However, there is limited understanding of their efficacy in triple negative breast cancer (TNBC). Therefore, there is an urgent need for proper patient stratification as well as relevant markers of sensitivity and resistance to CDK4/6 inhibitors. To address this, we performed a genome-wide loss-of-function CRISPR screen using palbociclib as a selection pressure to identify markers of sensitivity for CDK4/6is. The advent of CRISPR technology use in eukaryotic cells has revolutionized the way forward genetic screens are performed to answer biological questions, and large-scale in vitro CRISPR screens have been instrumental in identifying common essential genes [40–42] and new markers of drug sensitivity or resistance *in vitro* [43–45]*. *In vivo CRISPR screens are considered superior models, as they better recapitulate and more closely resemble the patient 3D tumor microenvironment [46, 47]. Our screen was performed in vivo to increase the translatability and clinical relevance of the results by better modeling the tumorigenic process.

Using GSEA, we cross-referenced our screening results with existing palbociclib sensitivity data from a panel of 38 breast cancer cell lines. This allowed us to validate that our screening results in TNBC were indeed viable in the larger context of other subtypes of breast cancer, including the well-established HR + /HER2- subtype. Our prioritization strategy notably attributed certain cell lines typifying the classically ‘CDK4/6 inhibitor-resistant’ phenotype to the ‘palbociclib more sensitive’ subgroup, paving the way for further studies to re-evaluate the criteria for choosing potential recipients of palbociclib treatment. Of note, past studies have often excluded TNBC on the basis of HR negativity, but, as witnessed here, other markers used together or alone could better predict the response to CDK4/6i treatment. Our screen identified several hundred candidate genes associated with sensitivity to palbociclib. Eight of the eight top candidate genes identified in our screen were found to mediate the loss of sensitivity to palbociclib, highlighting the robustness of our screening and hit prioritization approaches. Interestingly, 4/8 of our top targets (*TGFB3, ITGB6, BAMBI, PDGFB*) belong to the TGFβ signalling pathway, highlighting this pathway as an important regulator of the palbociclib response in TNBC.

Using available clinical trial data for ER + /HER2- BC patients with known clinical outcomes following palbociclib treatment (NeoPalAna) [28], we found that low expression of the top two validated genes, *SLC40A1* and *TGFB3*, correlated with resistance to palbociclib. This correlation validates the applicability of our results generated in a TNBC model, albeit Rb + , to other subtypes of breast cancer, namely ER + /HER2- breast cancer. This is also supported by the GSEA results. Ultimately, this reflects the usefulness of such screens in identifying clinically predictive molecular markers of responses to therapy in the future. These findings are especially relevant, given that the current predictive markers of response to CDK4/6 inhibitors are not foolproof. Markers, such as the presence of ER, are used as inclusion criteria in clinical trials for breast cancer and fail to reliably translate into meaningful clinical outcomes for many patients. Indeed, 20% of ER + patients enrolled in the phase III PALOMA-3 trial evaluating palbociclib efficacy were initially refractory to treatment (PFS < 6 months). An additional 50% of patients developed resistance to palbociclib during the first 24 months of treatment [48].

We retained *TGFB3* because of its remarkable effect in mediating sensitivity to palbociclib in vivo and its clinical relevance in predicting palbociclib resistance in the trial dataset. Despite the scarcity of information regarding the role of TGFβ3 in tumorigenesis [31], its function in normal tissues is relatively well defined. TGFβ3 plays an important role in embryogenesis, wound healing, scarless injury repair, and tissue homeostasis. This, in fact, led to the enrolment of recombinant human TGFβ3 (avotermin) in several phase I and II clinical trials for the prophylactic treatment of tissue scarring of the skin [31, 34]. Notably, TGFβ3 distinguishes its anti-scarring role from TGFβ1 and TGFβ2’s pro-scarring effects [34]. No safety concerns were raised before the termination of trials due to failure to show efficacy in phase III trials (possibly due to a change in the standard used to assay avotermin dosage, which ultimately led to much lower doses being used in phase III trials) [49]. In normal mammary tissue, it has been shown that TGFβ3 expression is increased during pregnancy, falling during lactation and peaking after weaning, during mammary gland involution. The massive induction of TGFβ3 after lactation, during mammary gland involution, contributes to the striking difference seen in expression levels as compared to TGFβ1 and TGFβ2 at this time [50–52]. TGFβ3’s distinct role in wound healing may explain how TGFβ3 relates to the tumorigenic process after mammary gland involution. Indeed, a parallel between mammary gland involution and tissue remodeling can be proposed; where TGFβ3, as opposed to TGFβ1 and TGFβ2, limits stromal activation associated with tissue scarring and pro-tumorigenic properties in this context [53]. In fact, in general breast cancer datasets, TGFβ3 seems to be protective against breast cancer [53]. Consistent with this, our results clearly highlight recTGFβ3 as a potential new combination treatment for patients with breast cancer receiving palbociclib.

To explore the predictive biomarker potential and clinical relevance of TGFβ3, we used the CRISPR activation system to overexpress endogenous TGFβ3 in TNBC tumors. We found that the anti-tumor effects of palbociclib were potentiated in *TGFB3*-overexpressing tumors, highlighting the value of *TGFB3* in predicting palbociclib response in TNBC. Collectively, these results help demonstrate that better patient stratification, for example through the inclusion of patients with higher *TGFB3* levels, during clinical trial enrolment may allow for patients with classically ‘unresponsive’ tumors, such as TNBC, to benefit from CDK4/6is. Future studies are required to determine whether measurement of TGFβ3 in liquid biopsies, for example, is feasible. The identification of biomarkers could have wider implications and be especially useful, given the current efforts being made to test the efficacy of CDK4/6is in other types of cancers.

We found that recTGFβ3 significantly potentiated the palbociclib-mediated inhibitory effects on cell proliferation and tumor growth, highlighting the clinical potential of recTGFβ3/palbociclib combination therapy for TNBC. TGFβ signalling is known to affect treatment sensitivity in breast cancer [54–57]. Of note, suppression of the TGFβ signalling pathway has previously been associated with resistance to CDK4/6 inhibitors through an extracellular miRNA-mediated mechanism in ER + breast cancer [58]. It would be interesting to further investigate whether the synergy observed between TGFβ3 and palbociclib is observable in other cancer types in which palbociclib treatment is being studied.

TGFβ induces the expression of the INK4 family of CDK inhibitors, including p21^CIP1^ (p21) [38, 59]. It has been shown that CDK4/6 inhibitors, including palbociclib, selectively redistribute p21 from CDK4/cyclin D1 complexes to inhibit CDK2 activity [39]. The role of p21 in the CDK4/6 inhibitor mechanism of action is not yet well established, but numerous reports indicate that low levels of p21 do seem to contribute to resistance to CDK4/6 inhibitors [60–63]. A study by Dean and colleagues demonstrated that prolonged exposure of cells to CDK4/6 inhibition leads to loss of the CDKIs p21 and p27 at the protein level only – not at the transcript level – implying that posttranscriptional mechanisms were responsible for this loss [62]. This decrease in p21 protein level may be likened to the loss in Rb protein, but not mRNA, following CDK4/6 exposure. While Rb degradation appears in many studies to be proteasome-dependent, it is unclear whether this process is dependent on ubiquitination [64–68]. Thus, it cannot be excluded that Rb is degraded by multiple mechanisms. We demonstrate that basal p21 levels are significantly lower in palbociclib resistant cells at the mRNA level, and that treatment with TGFβ3 leads to a significant increase in both p21 mRNA and protein levels in this context. Further studies elucidating how p21 levels are decreased by CDK4/6 inhibition, and indeed how this may compare to decreased Rb levels would be valuable. We demonstrated that the synergy observed between TGFβ3 and palbociclib was largely achieved through a p21-dependent mechanism, whereby the addition of recTGFβ3 induces p21 expression, which we posit helps inhibit still-active CDK4/6/cyclin D1 and CDK2/cyclin E1 complexes (Fig. 6). This dependence on p21 to achieve synergy between recTGFβ3 treatment and palbociclib treatment is further illustrated by the fact that administration of TGFβ1 and TGFβ2, which are also known to induce p21 in a Smad-dependent manner, equally potentiated the palbociclib effect in vitro. It is possible that the knockout efficiency of the other two TGFβ isoforms was not sufficient to produce a functional reduction of palbociclib sensitivity in the CRISPR/Cas9 screen used to identify TGFβ3, explaining why these other two isoforms did not appear enriched in the screen. The demonstration that stronger anti-tumorigenic effects could be achieved upon treatment with both palbociclib and recTGFβ3 simultaneously in multiple TNBC cell lines is of clinical relevance, especially considering the low concentrations of palbociclib at which this was achieved. Using lower concentrations of palbociclib, while still achieving comparable or even stronger anti-tumor responses while TGFβ3 levels are elevated, could help prevent some of the associated on-target toxicity in patient [37].

Patients often begin CDK4/6i treatment and become resistant to therapy over time. To address whether TGFβ3 could resensitize cells that had become insensitive to palbociclib treatment over time, we generated a palbociclib-resistant cell line over four months, and then treated the cells with recTGFβ3. We found that not only could TGFβ3 resensitize cells to palbociclib, but the combined effect of both TGFβ3 and palbociclib was significantly greater than the effect of either agent alone. Combination treatment with TGFβ3 and palbociclib achieved a synergistic anti-proliferative effect, indicating that administration of recTGFβ3 could be a relevant therapeutic strategy in the context of acquired resistance to palbociclib over time.

Altogether, this study exploited the synthetic lethal interaction between CDK4/6 and TGFβ3 and defined a new combinatorial treatment for TNBC using CDK4/6i and recombinant human TGFβ3. In addition, our study highlights TGFβ3 as a predictive marker to inform patient stratification for palbociclib treatment in breast cancer, underscoring the robustness of in vivo genome-wide CRISPR screening approaches to identify actionable biomarkers of drug response.

## Materials and methods

### Experimental design

This study used a genome-wide CRISPR/Cas9 loss-of-function screen to reveal markers of sensitivity and resistance to palbociclib in a CDK4/6 inhibitor-sensitive TNBC model. SUM159PT TNBC cells were infected with a genome-wide CRISPR library and transplanted into NSG mice. Palbociclib was administered to mice as tumors grew, and tumors were extracted and sequenced. Biological and technical replicates were measured. The aim was to identify candidate genes which could predict sensitivity or resistance to palbociclib across all molecular types of breast cancer. Therefore, candidates identified by sequencing were cross-referenced with their respective expression levels in publicly available microarray data from 38 breast cancer cell lines which were categorized based on known sensitivity to palbociclib. Using GSEA, top candidate genes were determined, and validation was performed orthotopically in vivo in NSG mice with daily injections of palbociclib*.* Loss of TGFβ3 using an individual CRISPR knockout in SUM159 was shown to generate resistance to palbociclib. TGFβ3 was further explored for its role in mediating palbociclib resistance, and it was demonstrated that treating cells with recombinant human TGFβ3 synergized with palbociclib in vivo in another model of TNBC, using preformed orthotopic mammary tumors derived from MDA-MB-231. This was also shown in the context of multiple palbociclib-naïve and palbociclib-resistant TNBC cell lines, and found to be p21-dependent. All experiments were performed with a minimum of three biological replicates. Tumor volumes were measured blindly with a digital caliper. Tumors were always randomized into vehicle and treatment groups, before treatment began.

### Cell lines and cell culture

SUM159PT and SUM229PE were cultured in Ham’s F-12, 1X (WISENT INC.) containing 5% fetal bovine serum (FBS, Gibco), 5 µg/mL insulin and 1 µg/mL hydrocortisone. More information about these cell lines is available at Breast Cancer Cell Line Knowledge Base (www.sumlineknowledgebase.com). MDA-MB-231 and HEK293T were cultured in Dulbecco’s Modified Eagle Medium (DMEM, WISENT INC.) supplemented with 10% FBS (Gibco). Cell lines were routinely tested by the Diagnostic Laboratory of the Comparative Medicine and Animal Resources Centre (McGill University) and are mycoplasma negative.

### Generation of 159-R cell line

SUM159PT cells were initiated to palbociclib isethionate (MedChemExpress, HY-A0065) exposure at a low concentration (100 nM) of the drug. Cells were passaged before reaching confluence and treated with incrementally higher concentrations of palbociclib (+ 100 nM every week for 12 weeks). After Week 12, the concentration was increased to 2 µM and was increased by 1 µM each week until 5 µM was reached.

### Genome-wide library (GeCKOv2) infection and in vivo transplantation

Human genome-scale CRISPR knockout pooled library (GeCKOv2, Addgene plasmid #1,000,000,048) was amplified according to manufacturer’s instructions and as shown previously [17]. 3 × 10^6^ SUM159PT cells were seeded per well in 12-well plates and polybrene (8 μg/mL) (EMD Millipore Corp. #TR-1003-G) was added to complete medium. Cells were spin-infected with previously titered lentivirus (MOI 0.3–0.5) at 800 × g for 2 h at 32 °C. Cells were then incubated overnight and subsequently detached, pooled and seeded into T225 flasks. 24 h following infection, puromycin (2 µg/mL) (InvivoGen) was added to medium and cells underwent selection over 9 days. 3 × 10^7^ cells were then collected and frozen at -80 °C for subsequent genomic DNA extraction. For each replicate of the screen, 3 × 10^7^ cells were transplanted subcutaneously in 4 nod-scid gamma (NSG) mice. Seven days later, once tumors were palpable, 2 mice were assigned to each treatment group. The vehicle (75% saline + 25% Tween-80) or palbociclib isethionate (MedChemExpress, HY-A0065) (30 mg/kg) dissolved in the vehicle was administered intraperitoneally 5 days/week for 23 days. Mice were sacrificed once it was no longer ethical to continue the experiment, when vehicle tumors became too large (experiment endpoint) and tumors were then collected and frozen at -80 °C for subsequent genomic DNA extraction.

### Genomic DNA extraction

For each sample, 3 × 10^7^ cells (cell representation sample) or 200 mg mechanically grinded tumor tissue (tumor sample) was lysed in 6 mL of NK Lysis Buffer (50 mM Tris, 50 mM EDTA, 1% SDS, pH 8) and 30 μL of 20 mg/mL Proteinase K (Qiagen). Cell lysates were incubated at 55 °C for 1 h (cell pellet) and tumor tissue was incubated overnight. RNAse A (QIAGEN) was added (0.05 mg/mL) and samples were incubated at 37 °C for 30 min, and then on ice for 10 min. 2 mL of ice-cold 7.5 M ammonium acetate (Sigma) was added to each sample before samples were briefly vortexed and centrifuged (4000 × g for 10 min). Supernatants were collected and isopropanol was added for DNA precipitation. Samples were centrifuged and remaining pellets were washed in 70% cold ethanol and resuspended in 1 × TE Buffer.

### Library preparation and deep sequencing

Next generation sequencing library was generated by two-step PCR. All PCR reactions were performed using Herculase II Fusion DNA Polymerase (Agilent). PCR1 reactions were prepared by mixing 20 μL Herculase 5 × Buffer, 1 μL of 100 mM dNTP, 2.5 μL of Adapter Primer F, 2.5 μL of Adapter Primer R, 1 μL Herculase II Fusion Enzyme, 10 μg of gDNA and completing to 100 μL with PCR-grade water. After individual validation, PCR1 reactions were pooled and stored at − 20 °C. PCR2 reactions were prepared by mixing 20 μL Herculase 5 × Buffer, 1 μL of 100 mM dNTP, 2.5 μL of Adapter Primer F, 2.5 μL of Adapter Primer R, 1 μL Herculase II Fusion Enzyme, 5 μL of PCR1 amplicon and completing to 100 μL with PCR-grade water. Final PCR products were migrated on a 2% agarose gel, extracted and purified using the QIAquick PCR & Gel Cleanup Kit (QIAGEN). Samples were sequenced (20 million reads) at Génome Québec (https://www.genomequebec.com/).

### Data processing and bioinformatics

MAGeCK and MAGeCK-VISPR were used to perform read count mapping, normalization, quality control and to identify sgRNA/gene hits [69]. sgRNA enrichment profile was generated by filtering for sgRNAs with false discovery rate (FDR) < 0.05. sgRNAs with mean control reads < 10 were removed, to reduce the potential for false positive hits included in the profile. Non-targeting and miRNA-targeting sgRNAs were further excluded from the profile. Significant hits were selected on the basis of having one or more specific gRNA out of the 3 sgRNAs/target present in the library, using a false discovery rate cutoff of < 0.05. It was also ensured that for each significantly enriched sgRNA targeting a given gene, no other gRNA targeting this gene was found to be depleted.

### Gene set enrichment analysis

Palbociclib sensitivity data from Finn et al. was used to rank 38 breast cancer cell lines, generating two profiles of cell lines, ‘sensitive’ (palbociclib IC50 < median) and ‘resistant’ (palbociclib IC50 > median) [7]. Gene expression data from the 38 cell lines was obtained from Kao et al [19]. The gene set used for gene set enrichment analysis was composed of the genes encoded by the 205 sgRNAs enriched (FDR < 0.05) in the in vivo CRISPR screen.

## CRISPR individual knockout and CRISPR activation plasmid cloning

For generation of knockout constructs, lentiCRISPRv2 backbone vector was obtained as a gift from Feng Zhang (Addgene plasmid # 52,961). For generation of activation constructs, lentiSAMv2 (Addgene plasmid # 75,112) and lentiMPHv2 (Addgene plasmid # 89,308) were used. Oligonucleotide sequences for KO and SAM sgRNAs are listed in Supplementary Table 1.

### Genomic DNA cleavage assay

Genomic DNA cleavage detection assays were performed for each individual gene knockout using the GeneArt Genomic Cleavage Detection Kit (Invitrogen, cat. no. A24372) according to the manufacturer’s protocol. Briefly, 5 × 10^5^ knockout cells were harvested and lysed. Genomic DNA was extracted and the specific Cas9/sgRNA genetically modified region was PCR-amplified using primers listed in Supplementary Table 1. Insertions or deletions (indels) to the region of interest were then detected.

### In vivo orthotopic xenograft studies

For individual gene knockout or activation validation, transduced SUM159PT knockout or activation cells (1 × 10^6^/mouse) were diluted 1:1 in Matrigel (BD Bioscience) and then transplanted in the mammary fat pads of 8-week-old, female NSG mice. Tumors were measured with an electronic caliper three times per week and allowed to reach a maximum volume of approximately 1000 mm^3^ prior to euthanasia. Tumor volumes were calculated according to the following formula: [4/3 × π × (length/2) × (width/2)^2^]. For treatments with palbociclib and/or recombinant human TGFβ3 ligand, SUM159PT- or MDA-MB-231- derived tumors were allowed to grow for 3–4 weeks until palpable. Palbociclib isethionate was dissolved in 75% saline and 25% Tween 80 (Sigma-Aldrich, P1754) solution. Palbociclib was administered in 10 mg/kg or 30 mg/kg doses. Recombinant human TGFβ3 ligand (PeproTech, Inc, cat. no. 100-36E) was dissolved in 10 mM citric acid buffer with 0.1% BSA. TGFβ3 was administered in 2 µg/kg doses. Volumes of all solutions injected were adjusted based on individual weight of each mouse. All injections were intraperitoneal. In the case where mice received combination treatment, a 4 h delay between palbociclib and TGFβ3 injections was respected to reduce the potential for formulation interactions between the two treatments. All mice were housed and handled in accordance with the approved guidelines of the Canadian Council on Animal Care (CCAC) “Guide to the Care and Use of Experimental Animals”.

### In vivo lung colonization studies

Individual CRISPR-mediated knockouts were generated in SUM159PT cells, and 1 × 10^6^ cells were injected into the tail vein of NSG mice to allow for lung colonization. Mice were euthanized and lung tissue was collected. Lungs were fixed and stained in Bouin’s solution and metastatic lesions were manually counted.

### NeoPalAna clinical trial

The NeoPalAna phase II clinical trial evaluated the efficacy of neoadjuvant palbociclib + anastrazole treatment in stage II-III ER + primary breast cancer [28]. The trial enrolled 50 patients. Patients received anastrozole (1 mg, daily) alone for the first 28 days (cycle 0), after which palbociclib (125 mg, daily) was added to the treatment regimen, on day 1 of cycle 1 of treatment (C1D1). Tumor biopsies were collected at C1D1, and 14 days following the start of palbociclib treatment (C1D15). If complete cell cycle arrest (Ki67 > 2.7%) was not achieved by C1D15, patients were deemed ‘resistant’ to treatment.

### Quantitative PCR

Frozen tumor tissues (50 mg) were homogenized in 1 mL TriZOL Reagent, and extraction proceeded according to the manufacturer’s protocol. RNA was reverse-transcribed using M-MLV Reverse Transcriptase (Invitrogen). Real-time PCR was performed using SsoFast EvaGreen Supermix (Bio-Rad) on a Rotor-Gene 6000 PCR analyzer (Corbett).

### Immunohistochemistry and scoring

Tumors were fixed in 10% formalin for minimum of 24 h. Tissues were paraffin embedded before they were mounted on slides. Following deparaffinization and rehydration, slides were immersed in retrieval solution (sodium citrate 10 mM, pH 6.0 buffer). The slides were incubated in hydrogen peroxide blocks, followed by Ultra V Block. Slides were incubated with Ki67 antibody. Ultra-Vision LP Detection System HRP Polymer & DAB Plus Chromogen (ThermoFisher Scientific) was used for detection. The slides were scanned using Aperio ScanScope XT slide (Leica Biosystems). Quantification of Ki67-positive tumor cells was performed using the Aperio Positive Pixel Count algorithm.

### Cell proliferation assay

Cells were seeded on 96-well plates and treated with palbociclib isethionate and/or recTGFβ3 at the indicated concentrations in complete medium for 5–7 days. Cells were then washed with PBS and stained and fixed with a 0.5% crystal violet solution in 25% methanol for 20 min at room temperature. Cell proliferation was assessed by absorbance at 570 nm. The percentage growth inhibition was used to calculate synergy scores using SynergyFinder https://synergyfinder.fimm.fi/.

### shRNA knockdown

Scramble, p21-specific, Smad2-specific and Smad3-specific shRNA plasmids were purchased from Sigma. Transfer vectors were transfected into HEK293T cells along with packaging plasmids p.MD2G and psPAX2. Virus was collected and used to infect 4.5 × 10^5^ SUM159 or SUM159 palbociclib-resistant (159-R) cells previously seeded in 6-cm plates and left to attach overnight. Cells were puromycin-selected (2 µg/mL) for 48 h and seeded for downstream analysis.

### Immunoblotting

Total protein were extracted in ice-cold lysis buffer (50 mM Tris–HCl, 150 mM NaCl, 1% Triton X-100, 1 mM EDTA, 100 mM Na3VO4, 1 × protease inhibitor cocktail and 1 × PhosStop Phosphatase Inhibitor Cocktail (Roche), diluted in 5 × loading buffer and boiled at 95 °C for 5 min. Samples were separated by SDS-PAGE, transferred onto nitrocellulose before being assessed by immunoblotting with the indicated antibodies.

### Flow cytometry

For cell synchronization, cells were serum starved for 24 h. Cells were released from arrest by addition of complete medium including 5% FBS for 24 h. Cells were treated with indicated agent palbociclib alone (100 nM), recTGFb3 alone (100 pM) or a combination of both (100 nM palbociclib + 100 pM recTGFb3). For propidium iodide (PI) staining, cells were detached, centrifuged at low speed and then counted. Following fixation with 70% ethanol, cells were washed twice with 1 × PBS. 100 µg/mL RNAase A and 50 µg/mL PI in 1 × PBS was added to 1 × 10^6^ cells for 30 min at 37°C, and cells were analyzed using the *BD FACSCanto*™ II flow cytometer (BD Biosciences).

### Statistical analyses

Multiple groups were compared using regular, one-way ANOVA with Tukey’s multiple comparisons tests. Difference between two group means was analyzed using unpaired, two-sided t-tests, with Holm-Šídák correction for multiple comparisons when applicable. Kaplan–Meier survival was analyzed using the log-rank test and presented as hazard ratios with 95% confidence intervals. P-values were considered significant when *p* < 0.05.

### Supplementary Information

Supplementary file 1.

Supplementary file 2.

Supplementary file 3.

## Data Availability

The data generated in this study are available within the article and its supplementary data files.
