# Supplementary material for: Genome-wide in vivo CRISPR screen identifies TGFβ3 as actionable biomarker of palbociclib resistance in triple negative breast cancer
Source: Mol Cancer. 2024 Jun 3;23:118. doi: 10.1186/s12943-024-02029-4 (PMC11145857; doi:10.1186/s12943-024-02029-4)
Supplement: Supplementary file 1 [file 12943_2024_2029_MOESM1_ESM.docx]

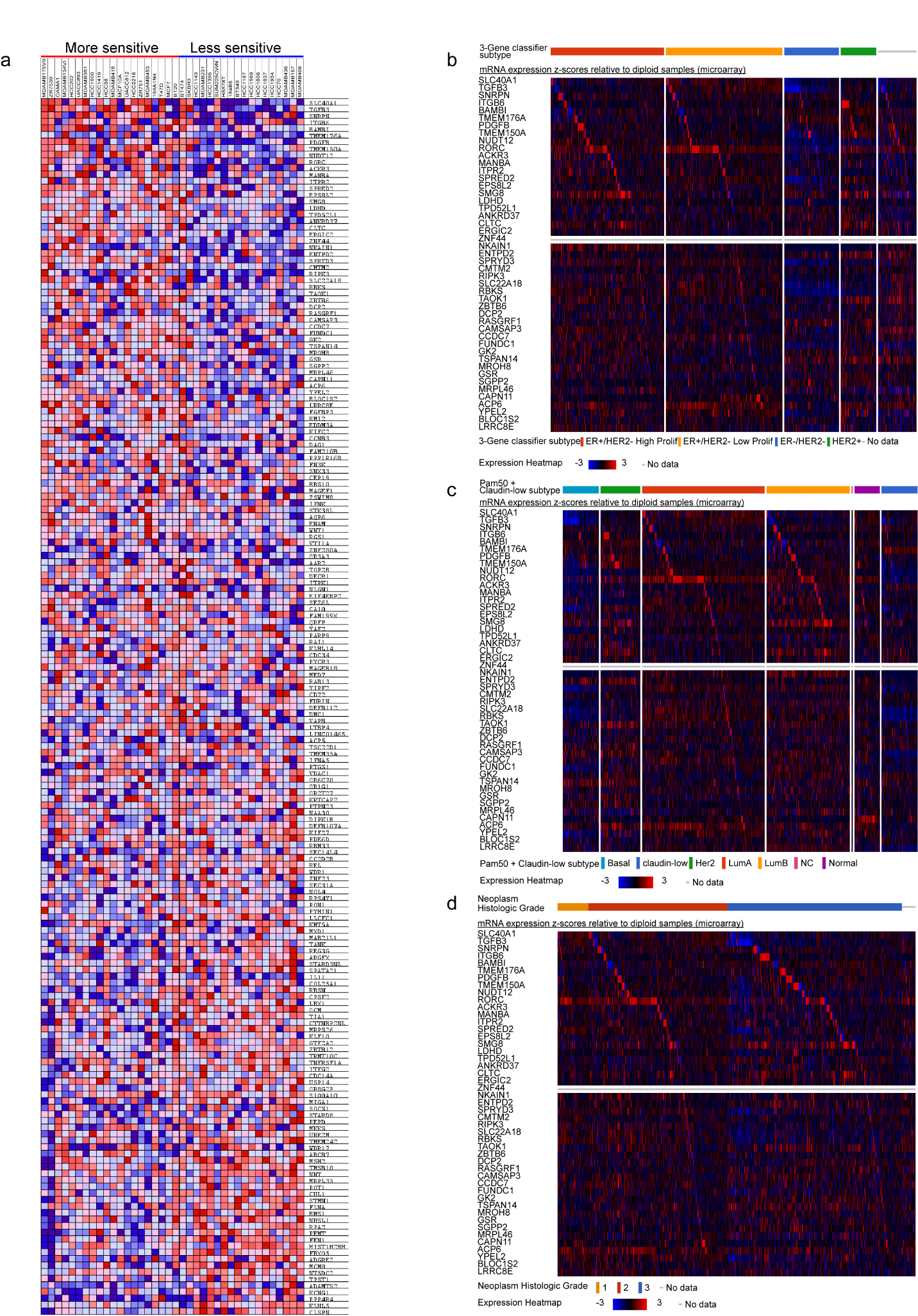


**Suppl. Fig. 1** ***In vivo* genome-wide CRISPR knockout screen in TNBC**. **a** The 205-gene set expression profile across panel of 38 breast cancer cell lines, following gene set enrichment analysis. Genes were ranked according to their level of representation in either group of cell lines (sensitive vs resistant) and the 205-gene set was found to be over-represented in the ‘palbociclib sensitive’ cell lines (FDR < 0.25). Source data are provided as a Source Data file. **b-d** mRNA expression of 47-gene core enrichment subset in 2,509 breast invasive carcinomas from the METABRIC dataset. **b** Oncoprint illustrating mRNA expression in tumors based on patient classification using ER and HER2 status. **c** mRNA expression based on Pam50 and claudin-low intrinsic subtypes. **d** mRNA expression based on tumor histologic grade.


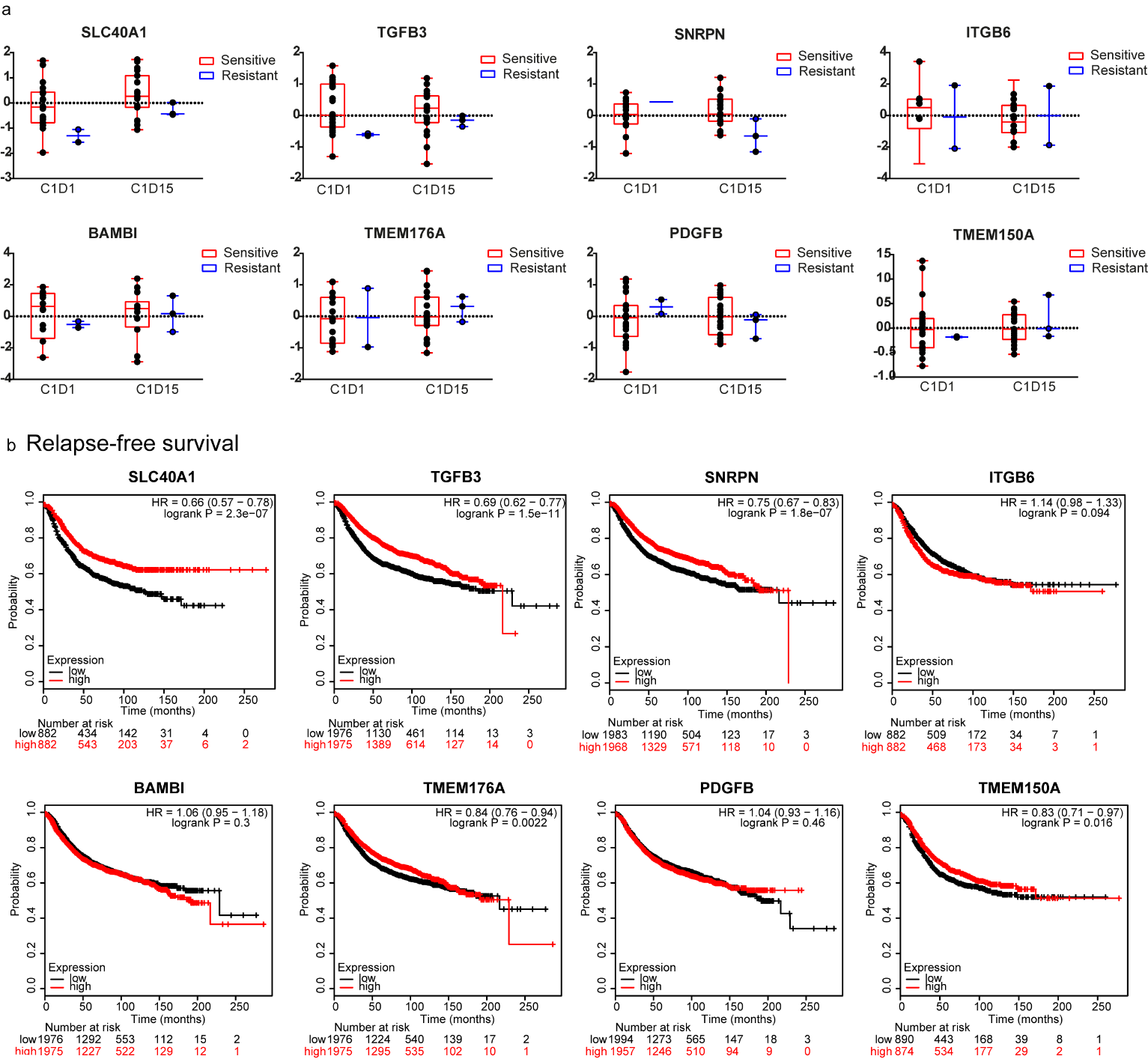


Suppl. Fig. 2 **Gene expression of top candidate genes in patients resistant to palbociclib.** **a** Patient data from the NeoPalAna phase II clinical trial evaluating palbociclib + anastrazole efficacy in stage II-III ER+ primary breast cancer. Patients received anastrozole alone for the first 28 days (cycle 0), after which palbociclib was added to the treatment regimen, on day 1 of cycle 1 of treatment (C1D1). Tumor biopsies were collected at C1D1, and 14 days following the start of palbociclib treatment (C1D15). If Ki67 > 2.7% at C1D15, patients were deemed ‘resistant’ to treatment. Box plots of tumor gene expression levels for each of the top candidate genes at two timepoints, C1D1 and C1D15. At each timepoint, data is presented by patient palbociclib response status (derived at C1D15 and applied retroactively to C1D1): sensitive (red) or resistant (blue). Topmost and bottommost ‘whiskers’ graph maximum and minimum values, respectively. Box outlines 25^th^ and 75^th^ percentiles, with midline inside box graphing the median value. **b** Kaplan-Meier analysis of relapse-free survival (RFS) outcomes based on mRNA gene expression of the top candidate genes across tumors of all breast cancer subtypes using KM Plotter. Patients were split by median. Hazard ratio is presented with 95% confidence intervals, and significance was calculated using log-rank test, *p*-value * <0.05.


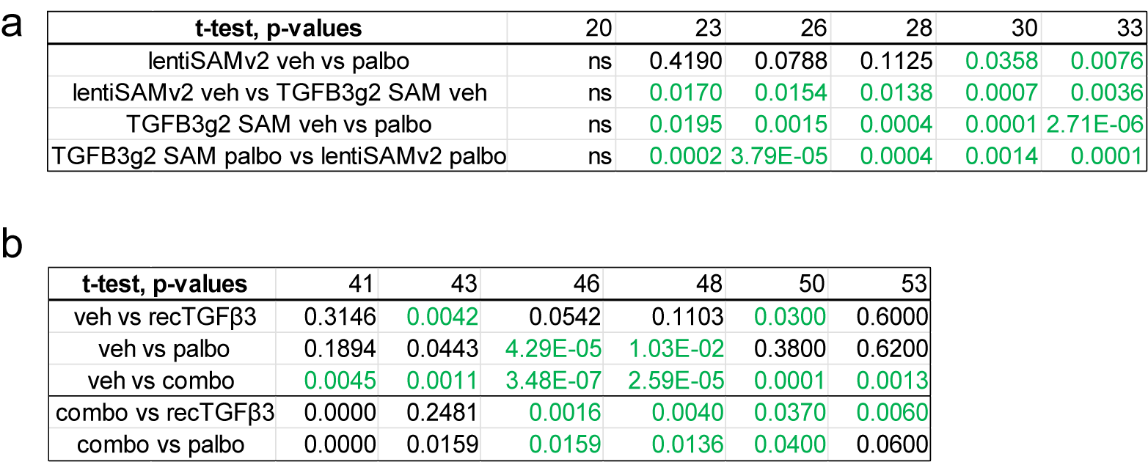


Suppl. Fig. 3 **Statistical significance of *in vivo* experiments. a** Table indicates significance for each group comparison at each time point. Significance was calculated using two-sided, unpaired t-test, *p*-value ns. = non-significant, green <0.05. b Table indicates significance for each group comparison at indicated time points. Significance was calculated using two-sided, unpaired t-test, *p*-value ns. = non-significant, green <0.05

**
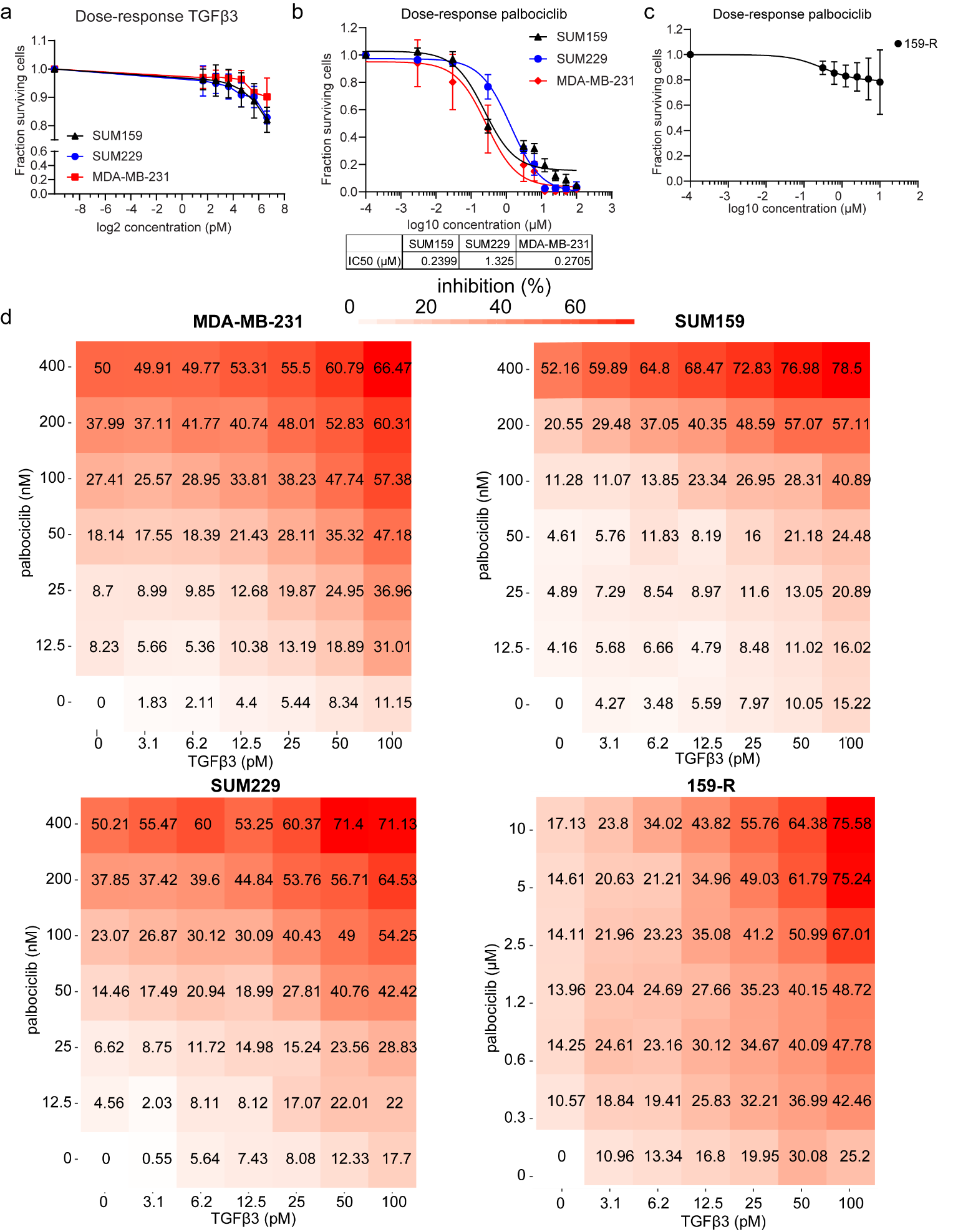
**Suppl. Fig. 4 **Combination of recombinant TGFβ3 and palbociclib synergistically inhibits TNBC cell proliferation *in vitro*.** **a** Dose-response curve of recombinant human TGFβ3 in the TNBC cell lines SUM159PT, SUM229, MDA-MB-231. Data presented are mean ± SD. **b** Dose-response curve of palbociclib in the TNBC cell lines SUM159PT, SUM229, MDA-MB-231, with accompanying IC50 values. Data presented are mean ± SD. **c** Dose-response curve of palbociclib in 159-R. Data presented are mean ± SD. d Grids representing inhibition of each dose combination tested using recTGFβ3 and palbociclib. Within each square is the percentage of inhibition of cell proliferation induced upon the indicated pairwise treatment. Each grid presents the results for one cell line.


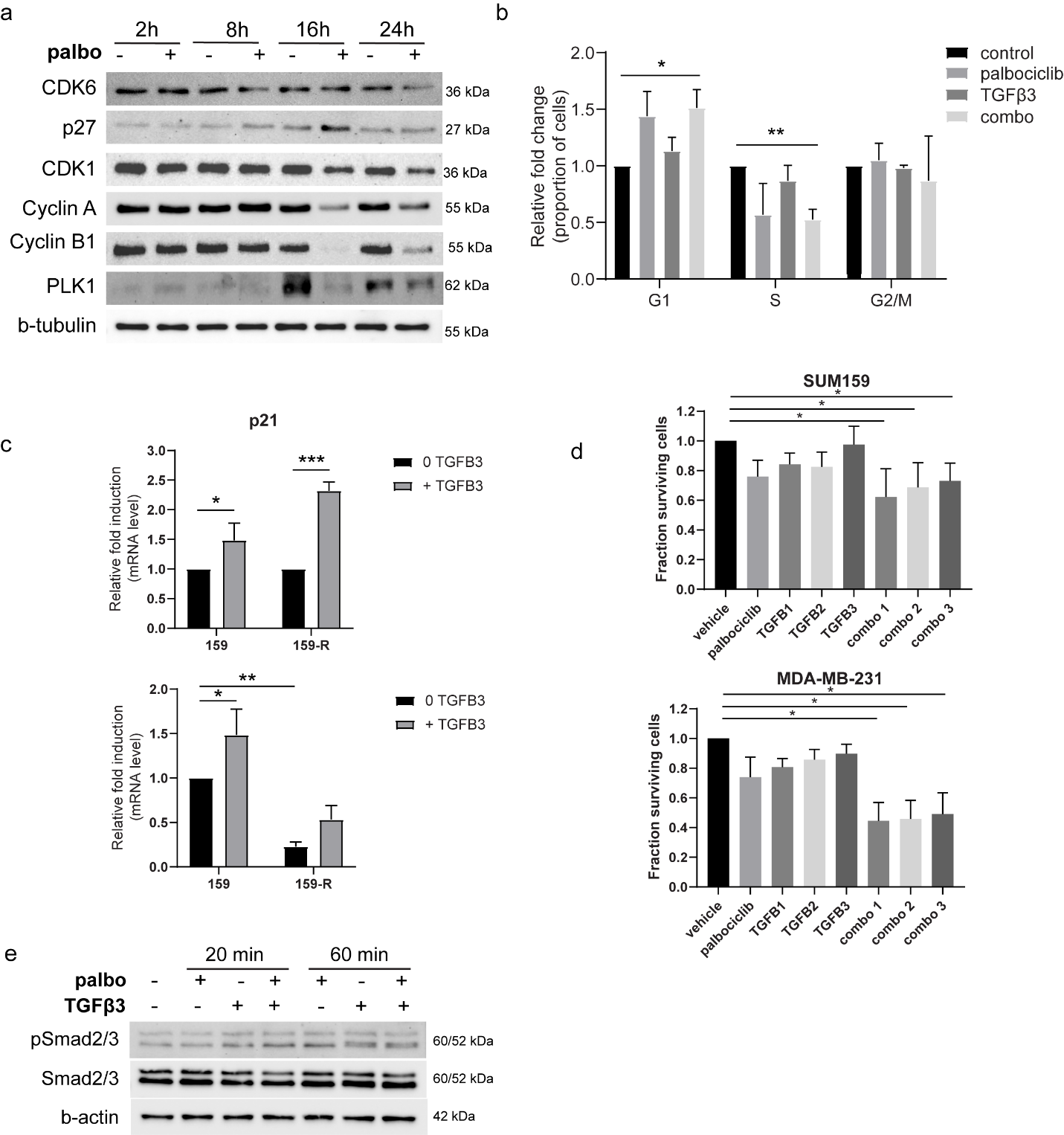


Suppl. Fig. 5 **TGFβ3 synergizes with palbociclib in a p21-dependent way. a** SUM159 cells were treated with palbociclib (100 nM) for 2h, 8h, 16h and 24h and levels of indicated proteins were assessed by immunoblotting with the appropriate antibodies. **b** Relative change in proportion of cells in indicated phase of the cell cycle, as compared to cells in the control (vehicle-treated) condition. Significance was calculated using multiple t-tests, corrected for multiple comparisons using the Holm-Šídák method, *p*-value * <0.05, ** <0.01. **c** mRNA expression levels of *CDKN1A* (p21) in SUM159 and 159-R (n = 3). Data are represented as mean ± standard deviation (SD). Significance was calculated using two-sided, unpaired t-test, *p*-value * <0.05. To compare means across cell lines, Significance was calculated using ordinary, one-way ANOVA with Tukey’s multiple comparisons test, *p*-value * <0.05, ** <0.01, *** <0.001. **d** SUM159 and MDA-MB-231 cells were treated with palbociclib (100 nM) alone or recTGFβ3 (100 pM) alone or a combination of both and the fraction of surviving was assessed using crystal violet staining (n=3). Data are represented as mean ± standard deviation (SD). Significance was calculated using ordinary, one-way ANOVA with Tukey’s multiple comparisons test, *p*-value * <0.05. **e** SUM159 cells were treated with palbociclib (100 nM) alone or recTGFβ3 (100 pM) alone or a combination of both for the indicated times and levels of phosphor-Smad2/3 and total Smad2/3 were assessed by immunoblotting.
