# Supplementary material for: Genome-wide in vivo CRISPR screen identifies TGFβ3 as actionable biomarker of palbociclib resistance in triple negative breast cancer
Source: Mol Cancer. 2024 Jun 3;23:118. doi: 10.1186/s12943-024-02029-4 (PMC11145857; doi:10.1186/s12943-024-02029-4)
Supplement: Supplementary file 2 [file 12943_2024_2029_MOESM2_ESM.pdf]

Fig 2a

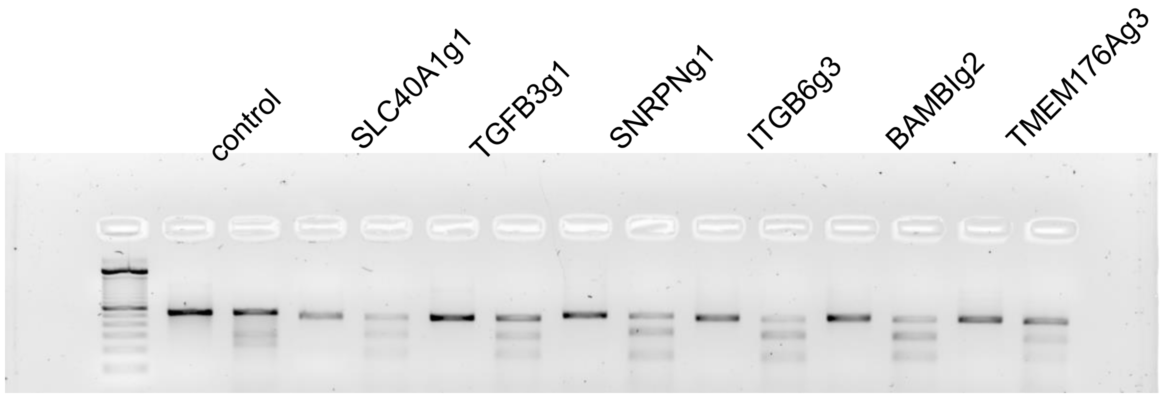
control
SLC40A1g1
TGFB3g1
SNRPNg1
ITGB6g3
BAMBIg2
TMEM176Ag3

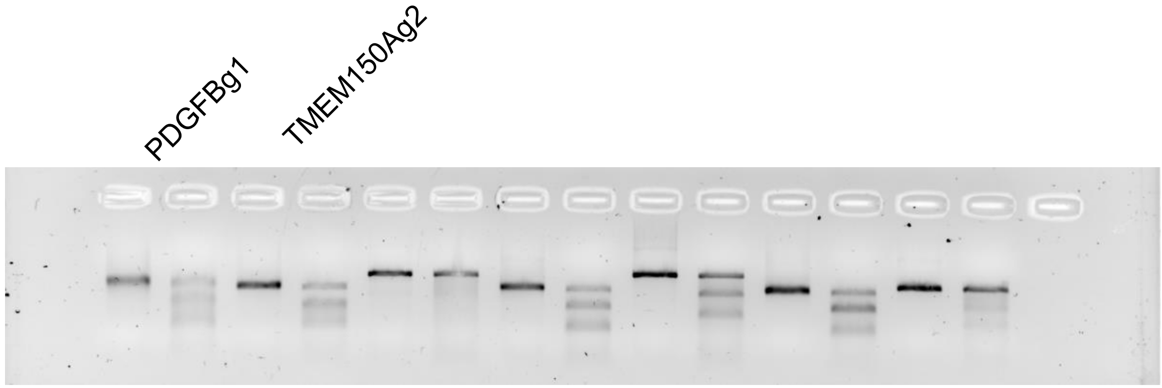
PDGFBg1
TMEM150Ag2

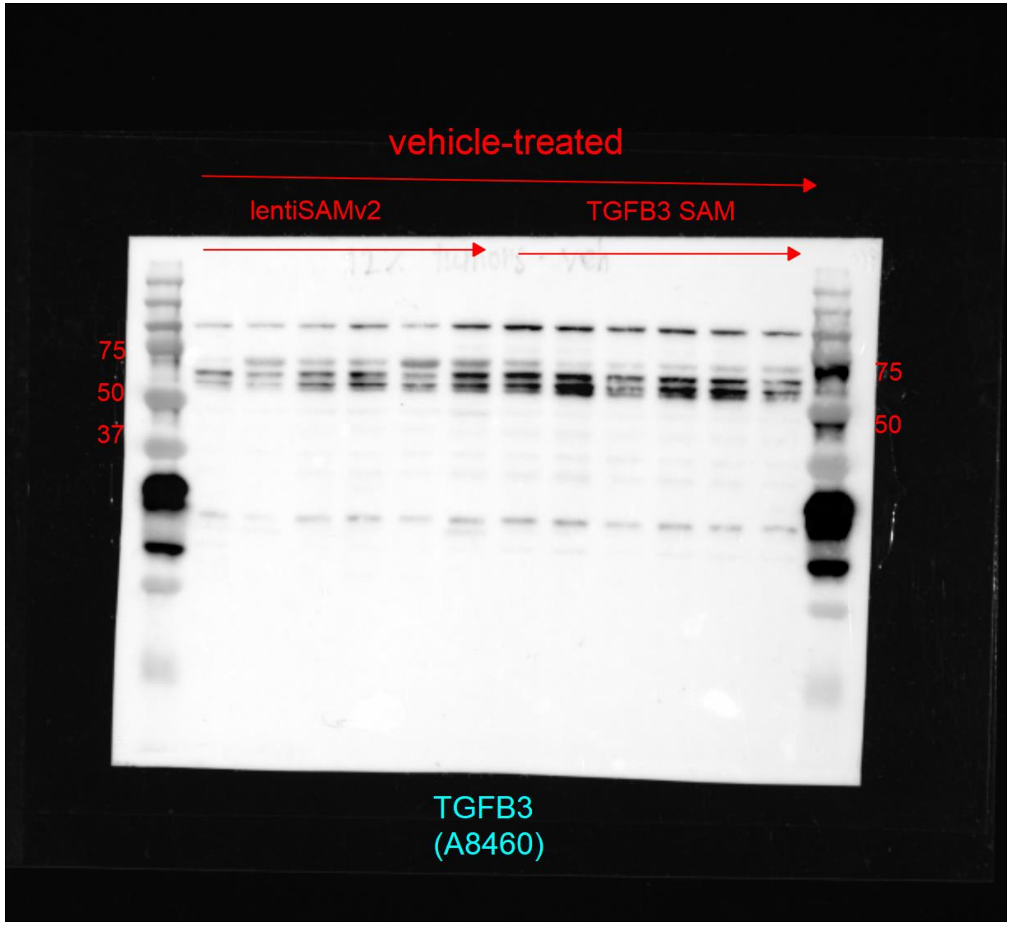
vehicle-treated
lentiSAMv2
TGFB3 SAM
75
50
37
75
50
TGFB3
(A8460)

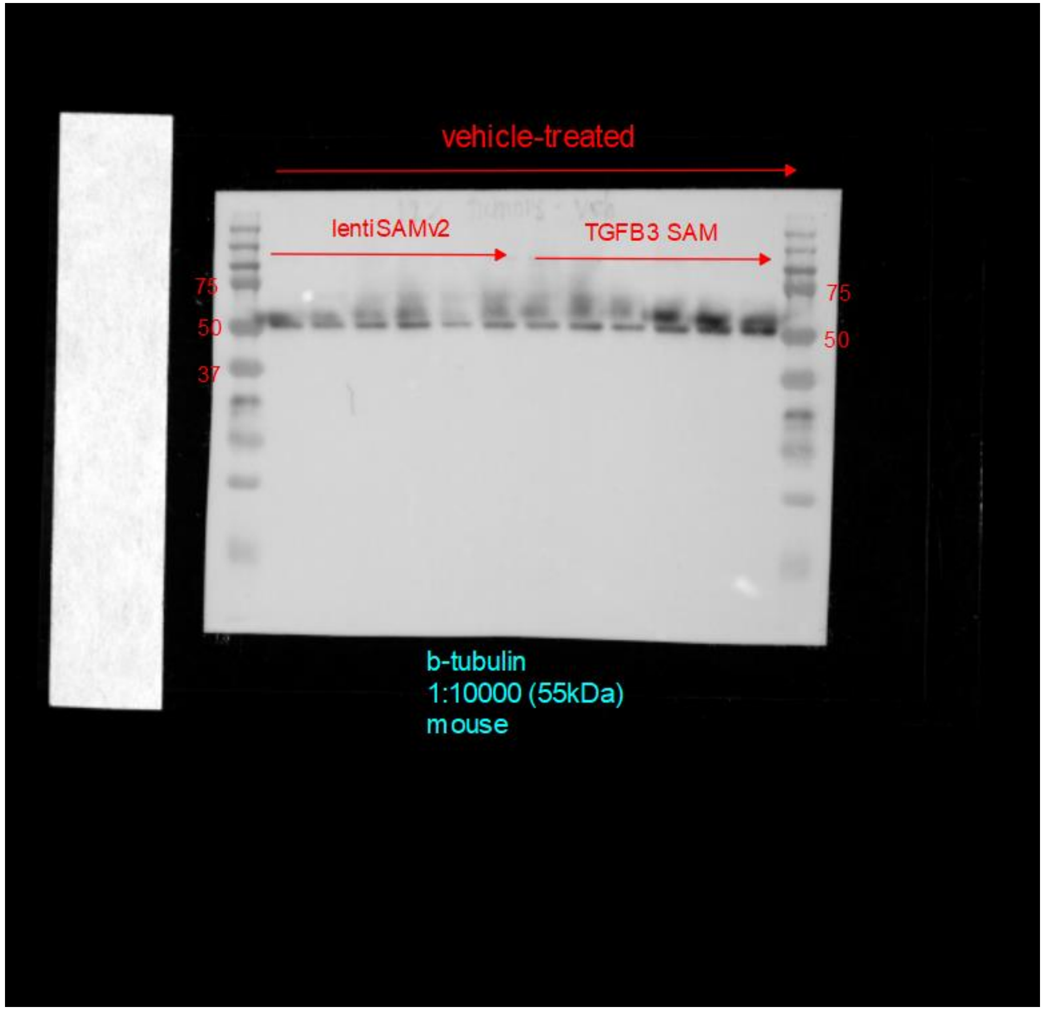
vehicle-treated
lentiSAMv2
TGFB3 SAM
75
50
37
75
50
b-tubulin
1:10000 (55kDa)
mouse

Fig 3f

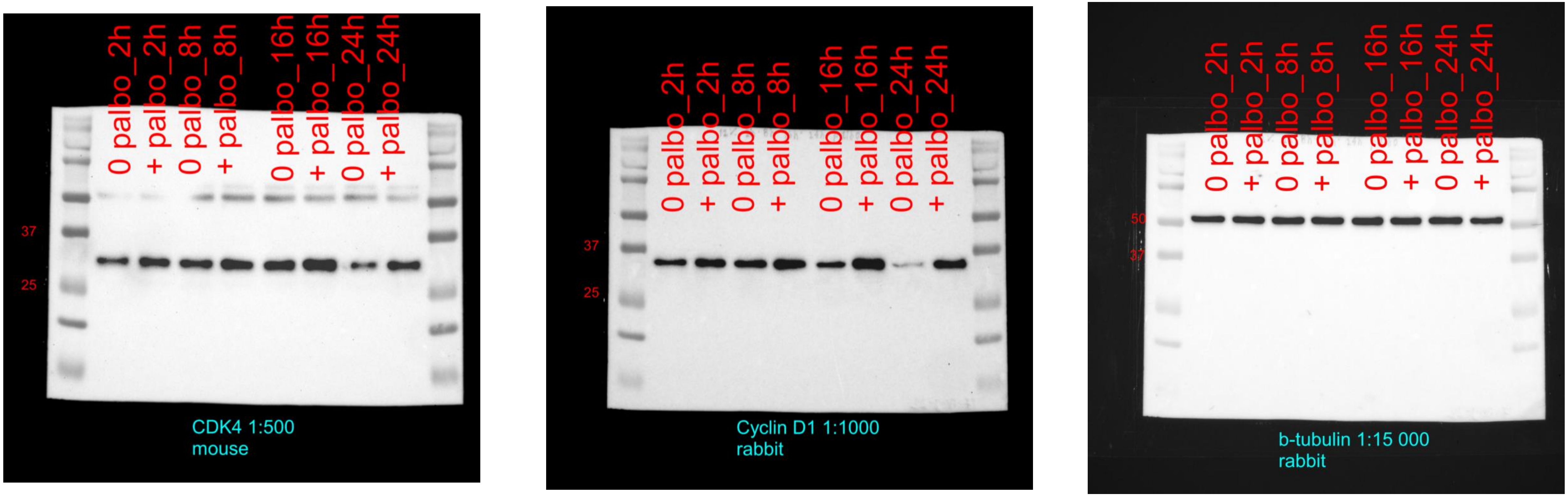
0 palbo_2h
+ palbo_2h
0 palbo_8h
+ palbo_8h
0 palbo_16h
+ palbo_16h
0 palbo_24h
+ palbo_24h
37
25
CDK4 1:500
mouse
0 palbo_2h
+ palbo_2h
0 palbo_8h
+ palbo_8h
0 palbo_16h
+ palbo_16h
0 palbo_24h
+ palbo_24h
37
25
Cyclin D1 1:1000
rabbit
0 palbo_2h
+ palbo_2h
0 palbo_8h
+ palbo_8h
0 palbo_16h
+ palbo_16h
0 palbo_24h
+ palbo_24h
50
37
b-tubulin 1:15 000
rabbit

Fig 5a

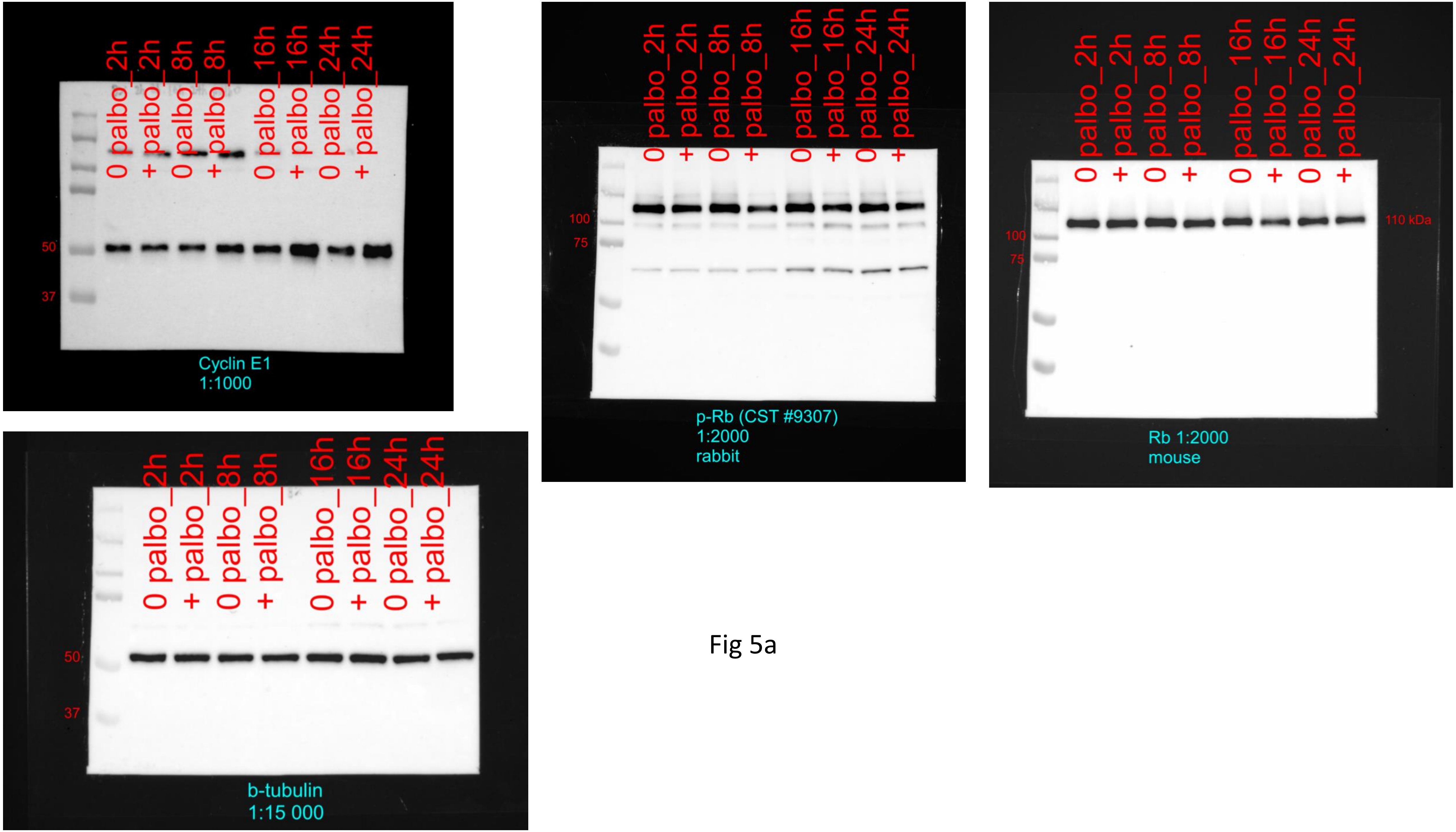
0 palbo_2h
+ palbo_2h
0 palbo_8h
+ palbo_8h
0 palbo_16h
+ palbo_16h
0 palbo_24h
+ palbo_24h
50
37
Cyclin E1
1:1000
100
75
p-Rb (CST #9307)
1:2000
rabbit
110 kDa
Rb 1:2000
mouse
b-tubulin
1:15 000

Fig 5a

Fig 5b

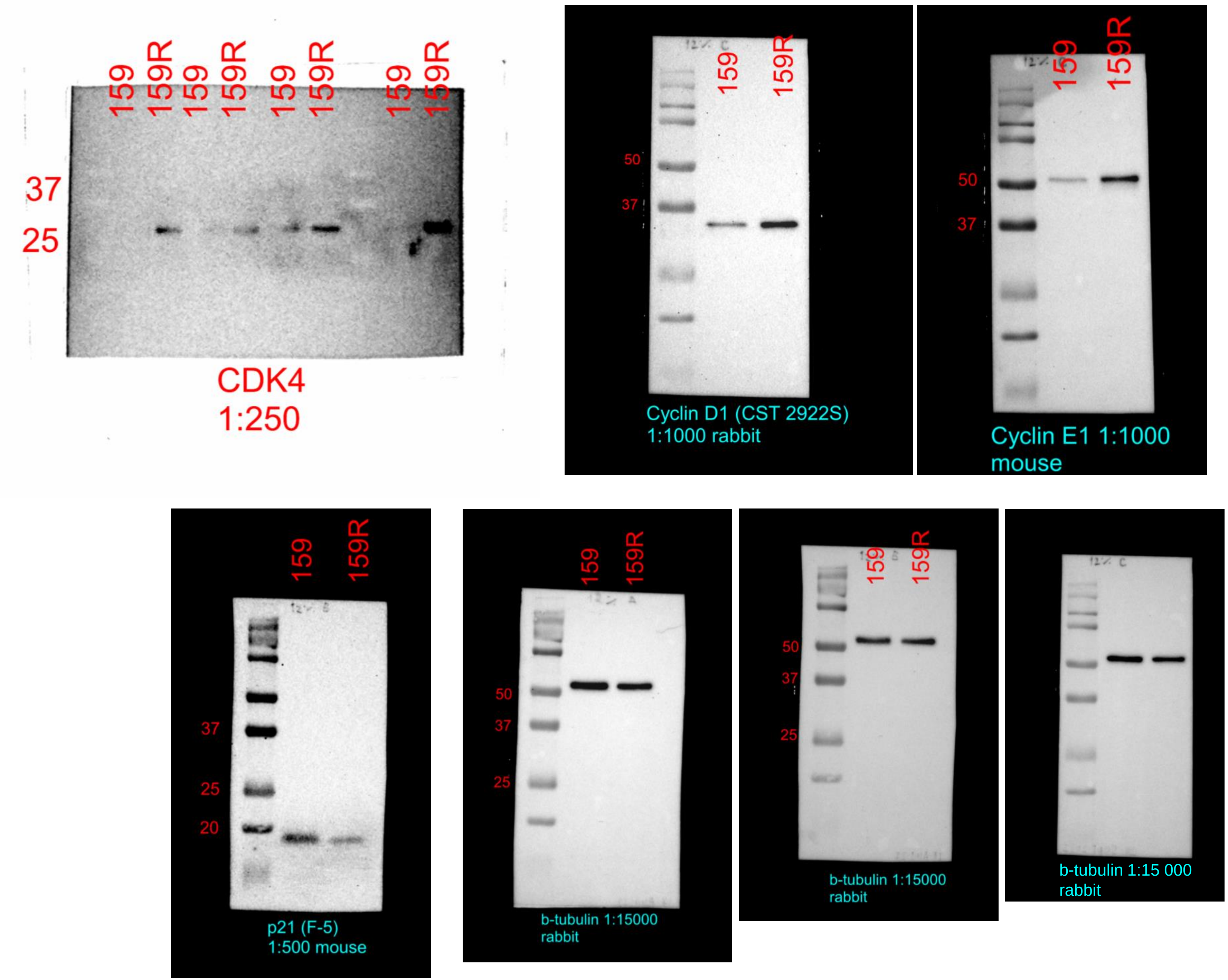

159
159R
159
159R
159
159R
159
159R
37
25
CDK4
1:250
159
159R
50
37
Cyclin D1 (CST 2922S)
1:1000 rabbit
159
159R
50
37
Cyclin E1 1:1000
mouse
159
159R
37
25
20
p21 (F-5)
1:500 mouse
159
159R
50
37
25
b-tubulin 1:15000
rabbit
159
159R
50
37
25
b-tubulin 1:15000
rabbit
b-tubulin 1:15 000
rabbit

Fig 5b

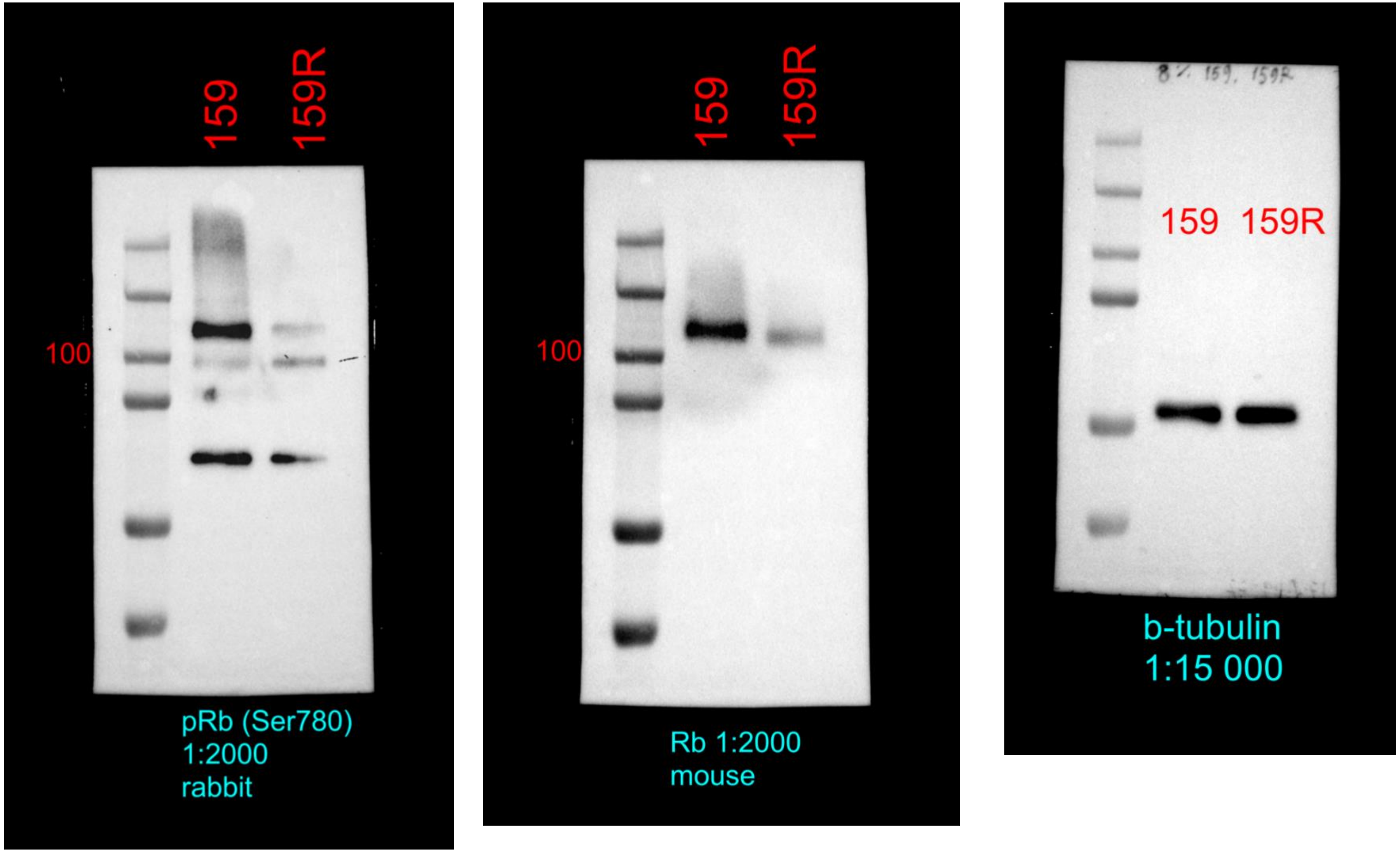
159
159R
100
pRb (Ser780)
1:2000
rabbit
159
159R
100
Rb 1:2000
mouse
159 159R
b-tubulin
1:15 000

Fig 5c

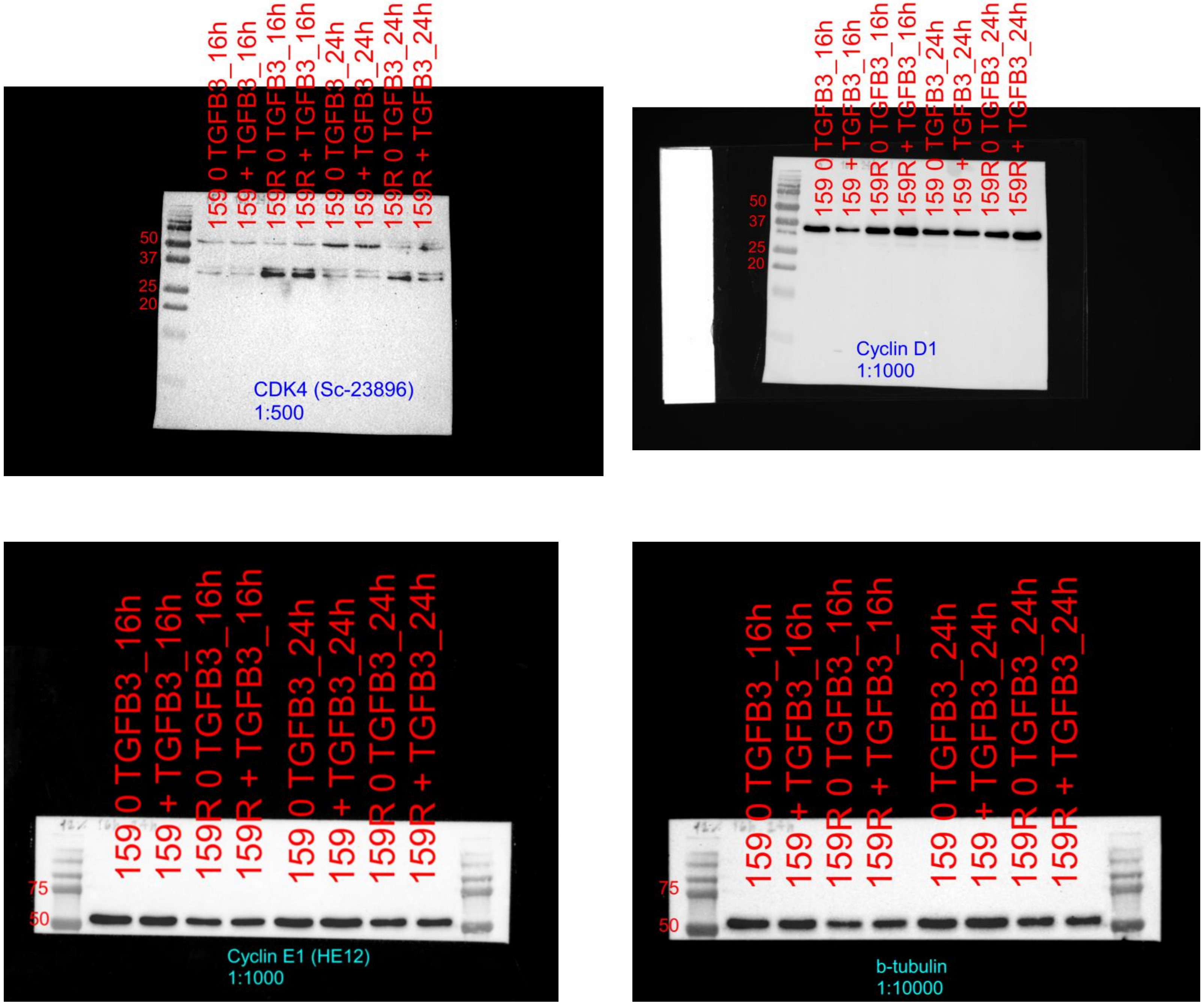
159 0 TGFB3_16h
159 + TGFB3_16h
159R 0 TGFB3_16h
159R + TGFB3_16h
159 0 TGFB3_24h
159 + TGFB3_24h
159R 0 TGFB3_24h
159R + TGFB3_24h
50
37
25
20
CDK4 (Sc-23896)
1:500
Cyclin D1
1:1000
75
Cyclin E1 (HE12)
1:1000
b-tubulin
1:10000

Fig 5c

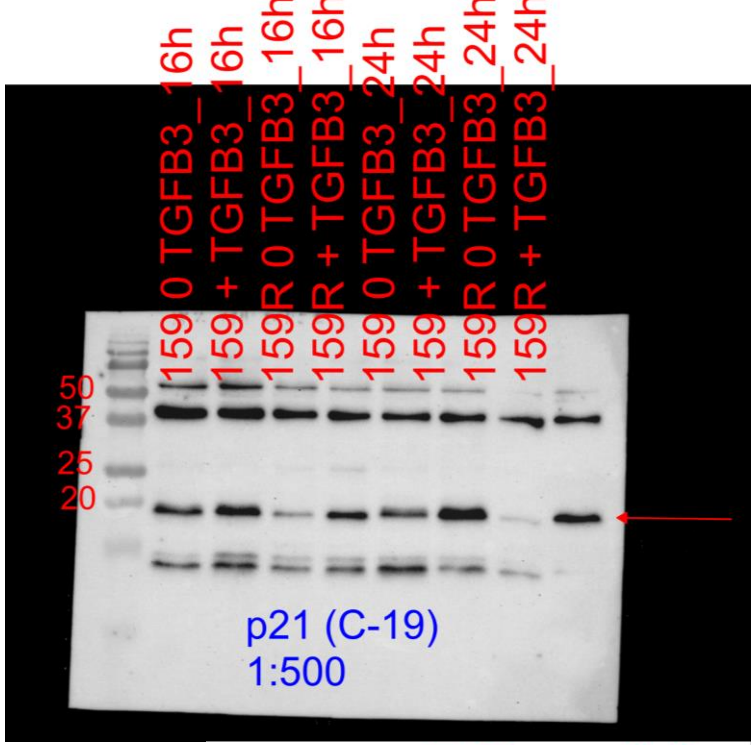
159 0 TGFB3_16h
159 + TGFB3_16h
159R 0 TGFB3_16h
159R + TGFB3_16h
159 0 TGFB3_24h
159 + TGFB3_24h
159R 0 TGFB3_24h
159R + TGFB3_24h
50
37
25
20
p21 (C-19)
1:500

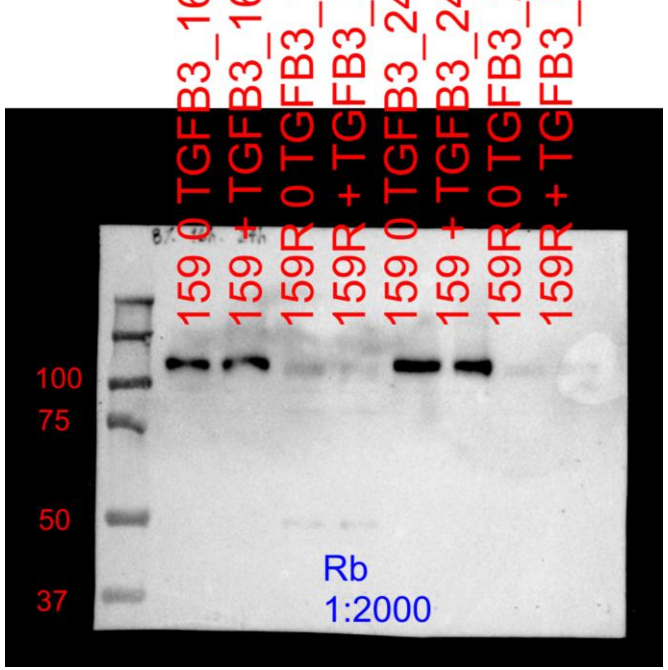
100
75
50
37
Rb
1:2000

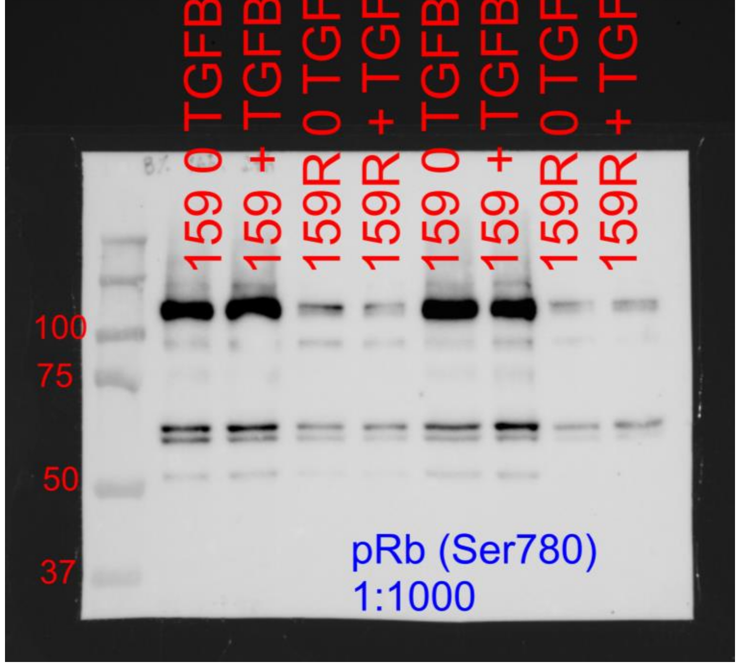
100
75
50
37
pRb (Ser780)
1:1000

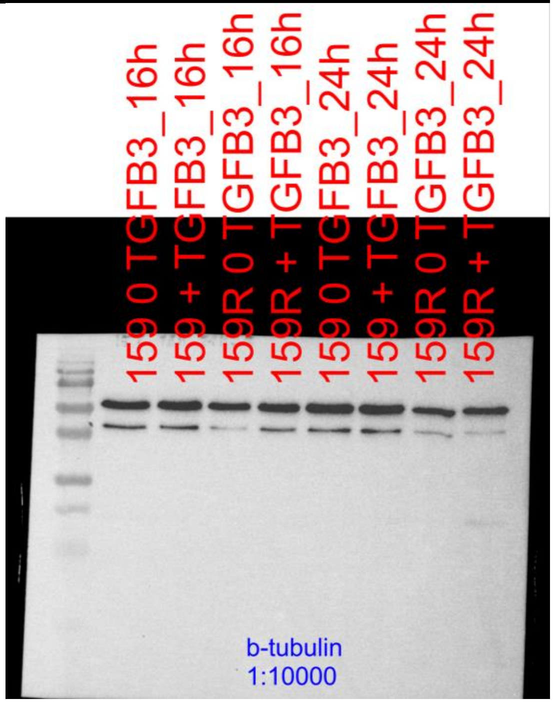
159 0 TGFB3_16h
159 + TGFB3_16h
159R 0 TGFB3_16h
159R + TGFB3_16h
159 0 TGFB3_24h
159 + TGFB3_24h
159R 0 TGFB3_24h
159R + TGFB3_24h
b-tubulin
1:10000

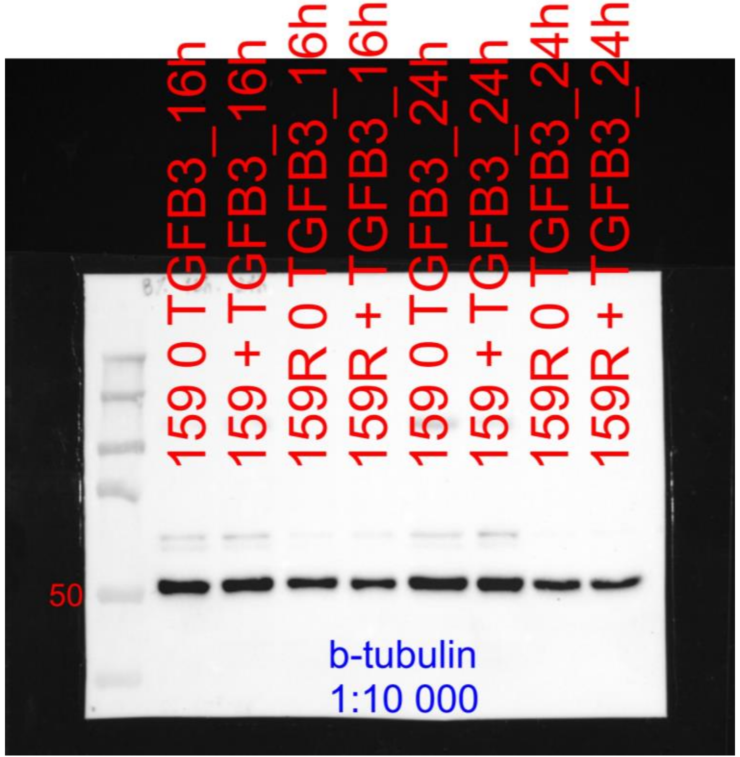
159 0 TGFB3_16h
159 + TGFB3_16h
159R 0 TGFB3_16h
159R + TGFB3_16h
159 0 TGFB3_24h
159 + TGFB3_24h
159R 0 TGFB3_24h
159R + TGFB3_24h
50
b-tubulin
1:10 000

Fig 5c

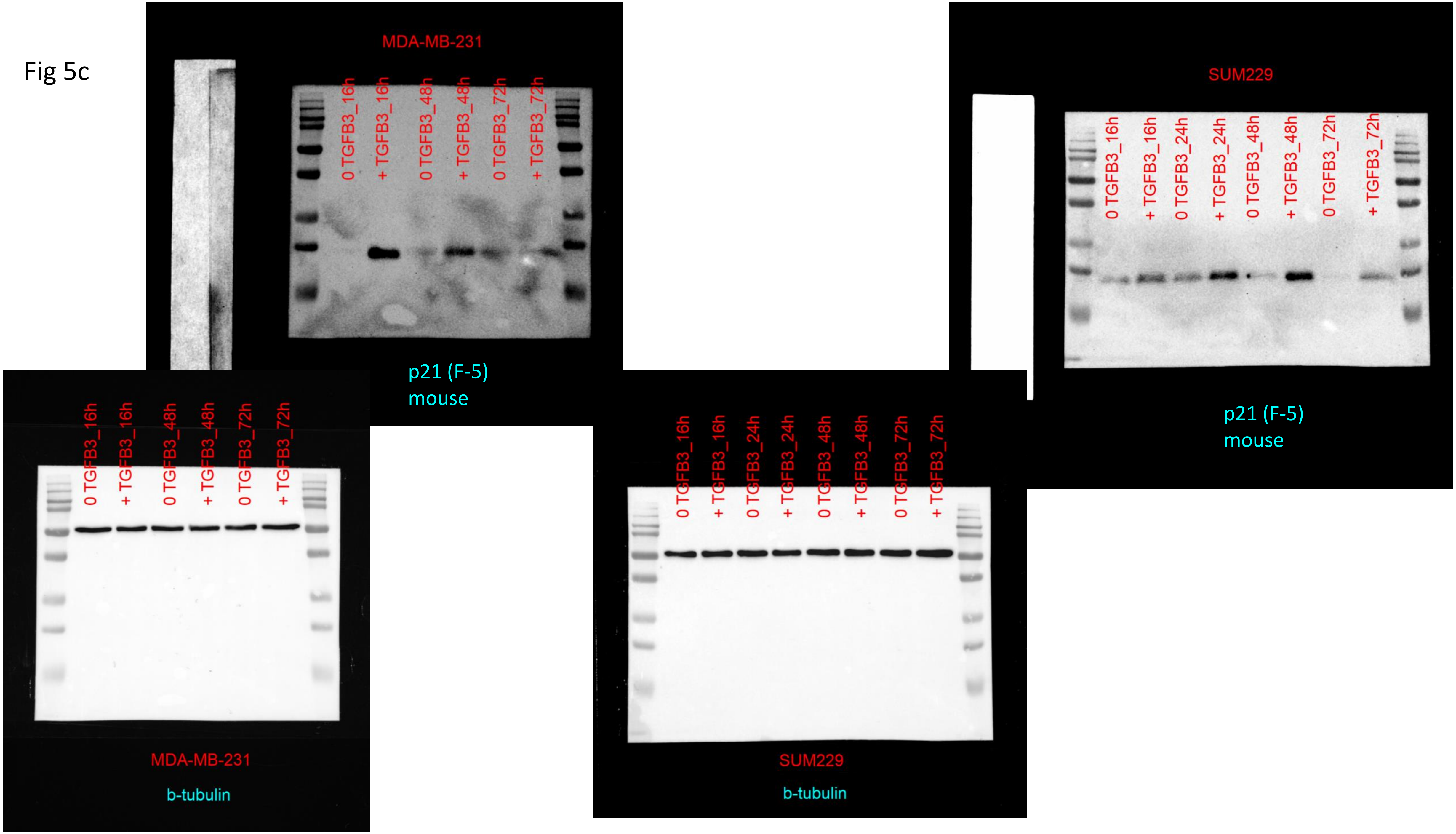
MDA-MB-231
0 TGFB3_16h
+ TGFB3_16h
0 TGFB3_48h
+ TGFB3_48h
0 TGFB3_72h
+ TGFB3_72h
p21 (F-5)
mouse
SUM229
0 TGFB3_16h
+ TGFB3_16h
0 TGFB3_24h
+ TGFB3_24h
0 TGFB3_48h
+ TGFB3_48h
0 TGFB3_72h
+ TGFB3_72h
p21 (F-5)
mouse
0 TGFB3_16h
+ TGFB3_16h
0 TGFB3_48h
+ TGFB3_48h
0 TGFB3_72h
+ TGFB3_72h
MDA-MB-231
b-tubulin
0 TGFB3_16h
+ TGFB3_16h
0 TGFB3_24h
+ TGFB3_24h
0 TGFB3_48h
+ TGFB3_48h
0 TGFB3_72h
+ TGFB3_72h
SUM229
b-tubulin

Fig 5d

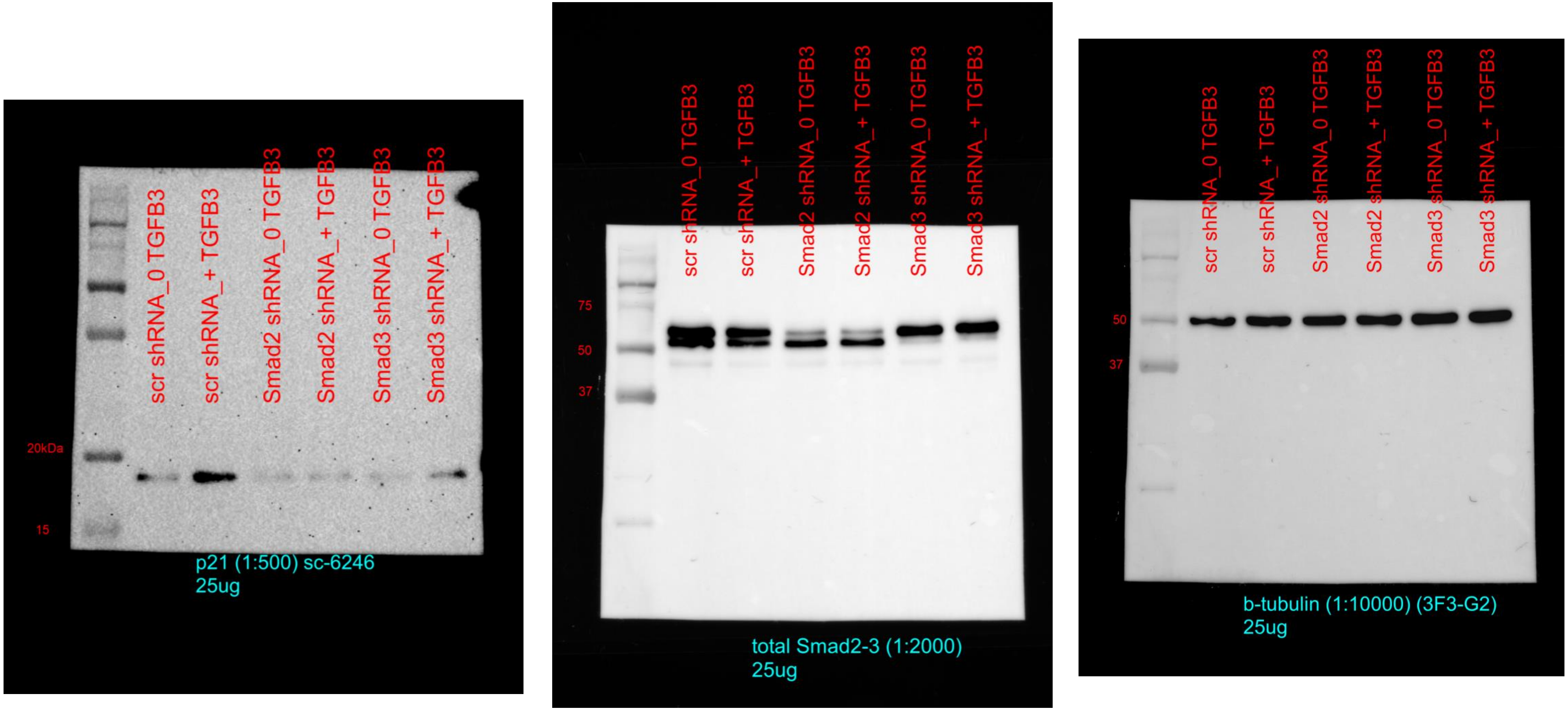
scr shRNA_0 TGFB3
scr shRNA_+ TGFB3
Smad2 shRNA_0 TGFB3
Smad2 shRNA_+ TGFB3
Smad3 shRNA_0 TGFB3
Smad3 shRNA_+ TGFB3
20kDa
15
p21 (1:500) sc-6246
25ug
75
50
37
total Smad2-3 (1:2000)
25ug
50
37
b-tubulin (1:10000) (3F3-G2)
25ug

Fig 5e

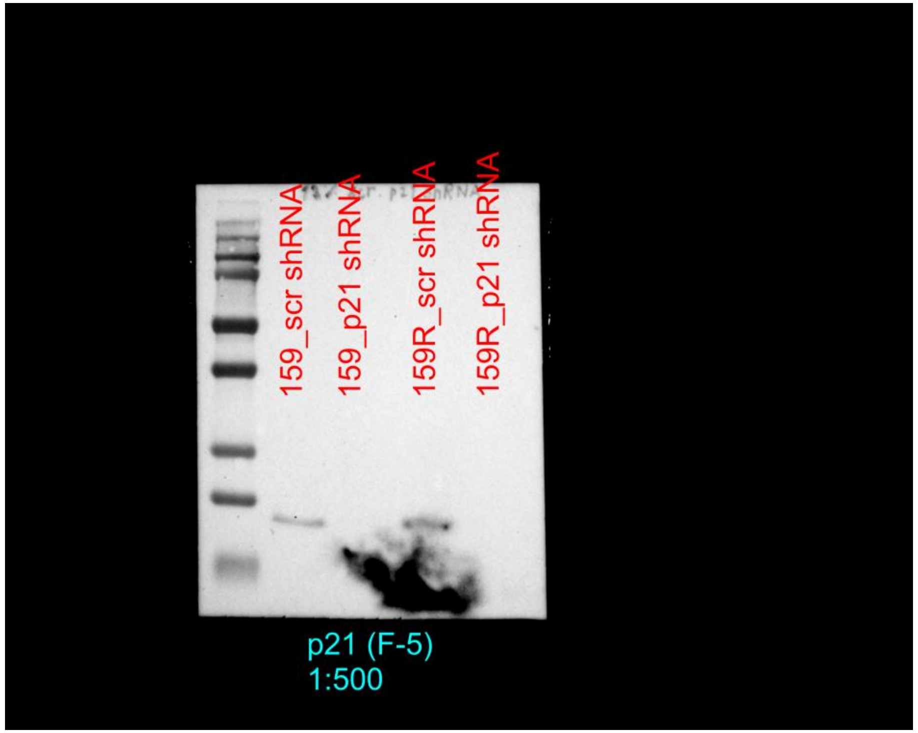
159_scr shRNA
159_p21 shRNA
159R_scr shRNA
159R_p21 shRNA
p21 (F-5)
1:500

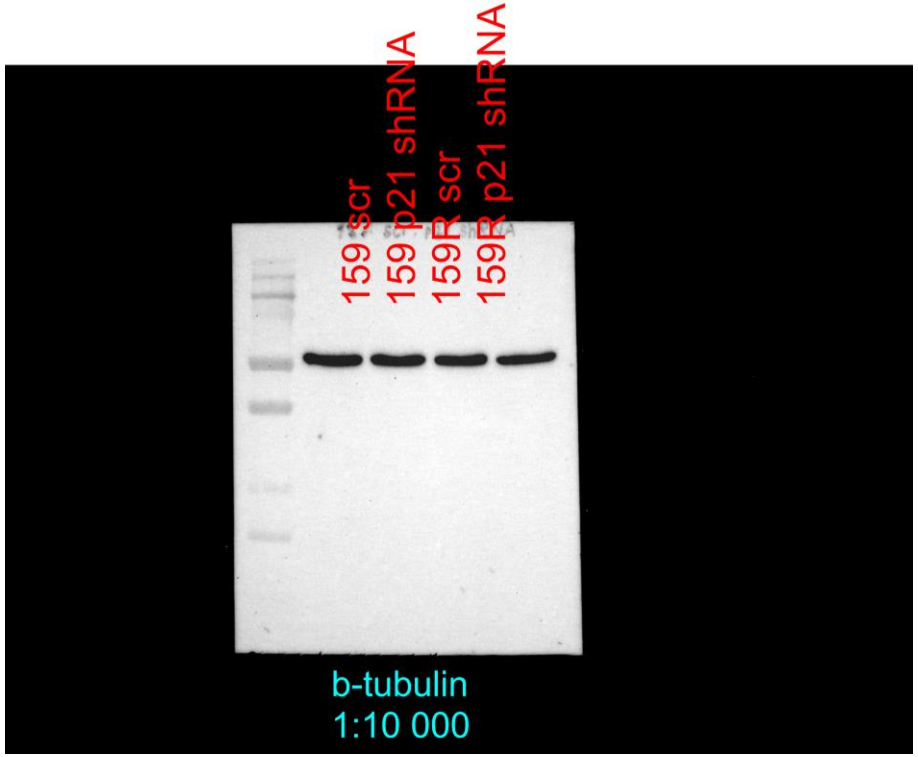
159 scr
159 p21 shRNA
159R scr
159R p21 shRNA
b-tubulin
1:10 000

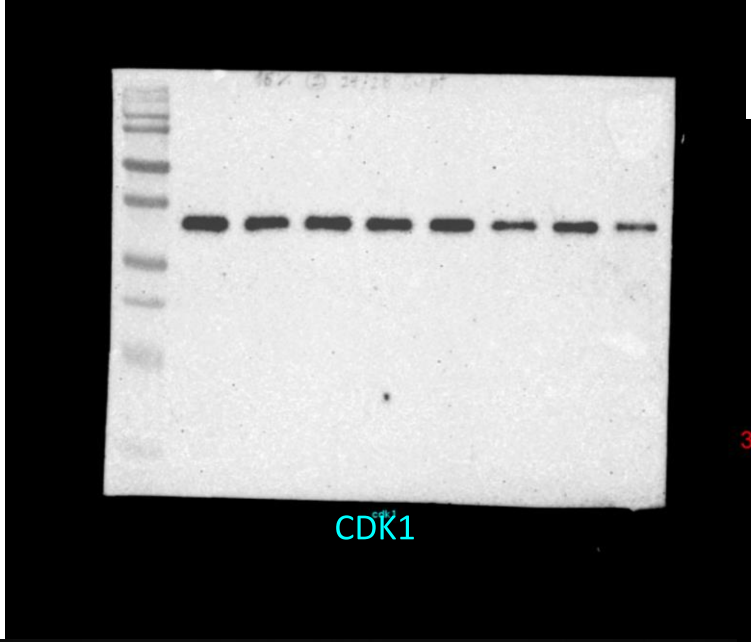
CDK1

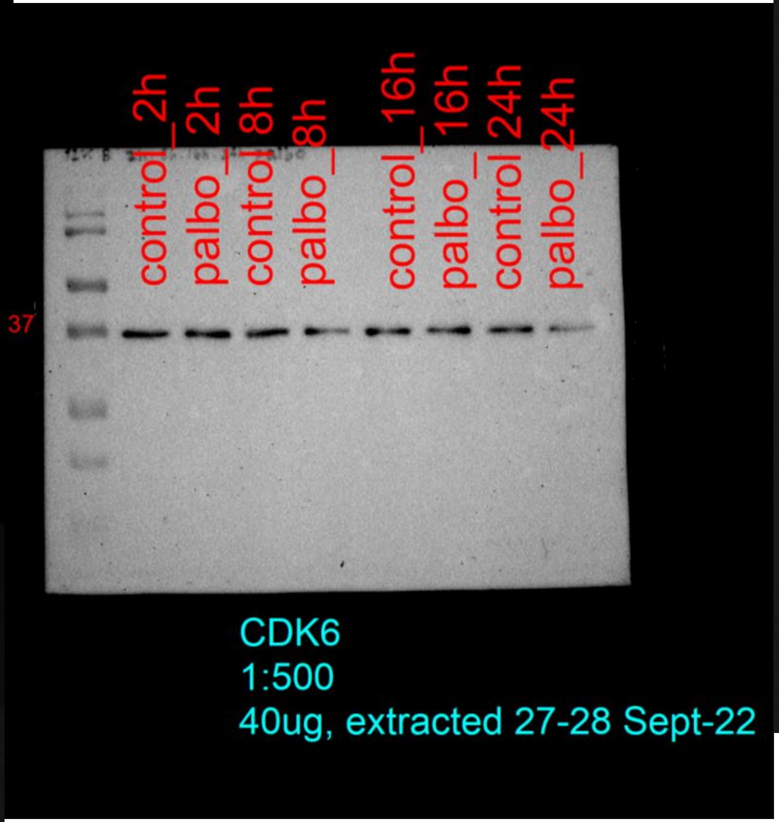
control_2h
palbo_2h
control_8h
palbo_8h
control_16h
palbo_16h
control_24h
palbo_24h
37
CDK6
1:500
40ug, extracted 27-28 Sept-22

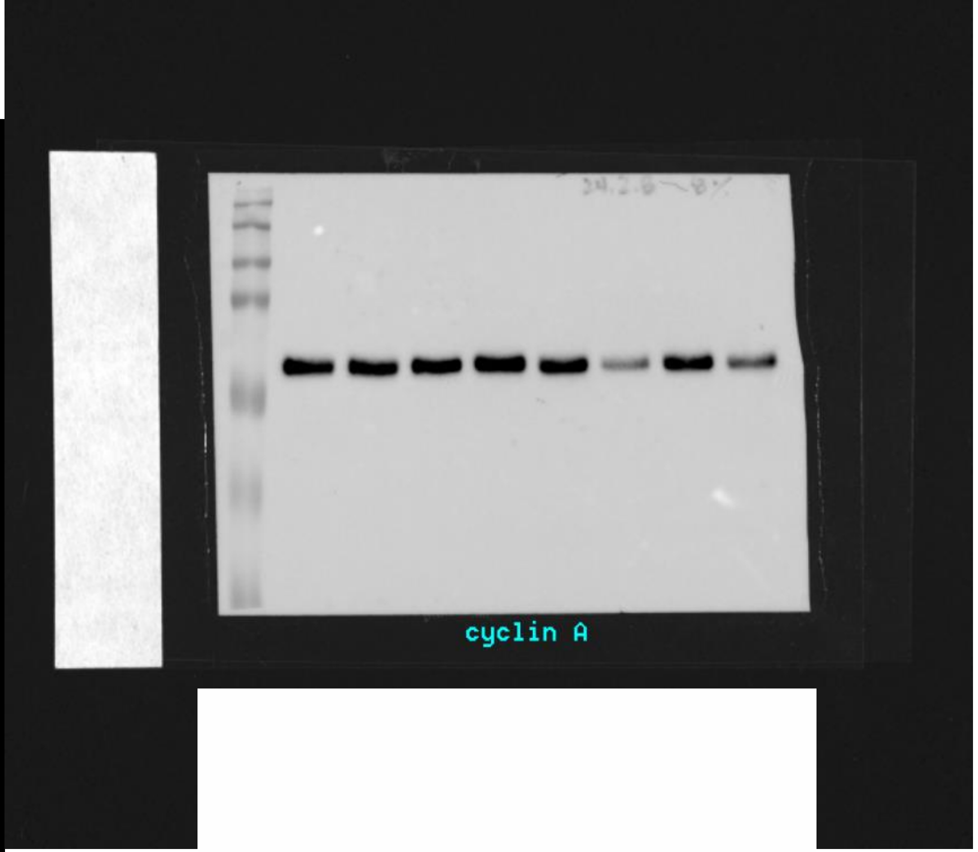
cyclin A

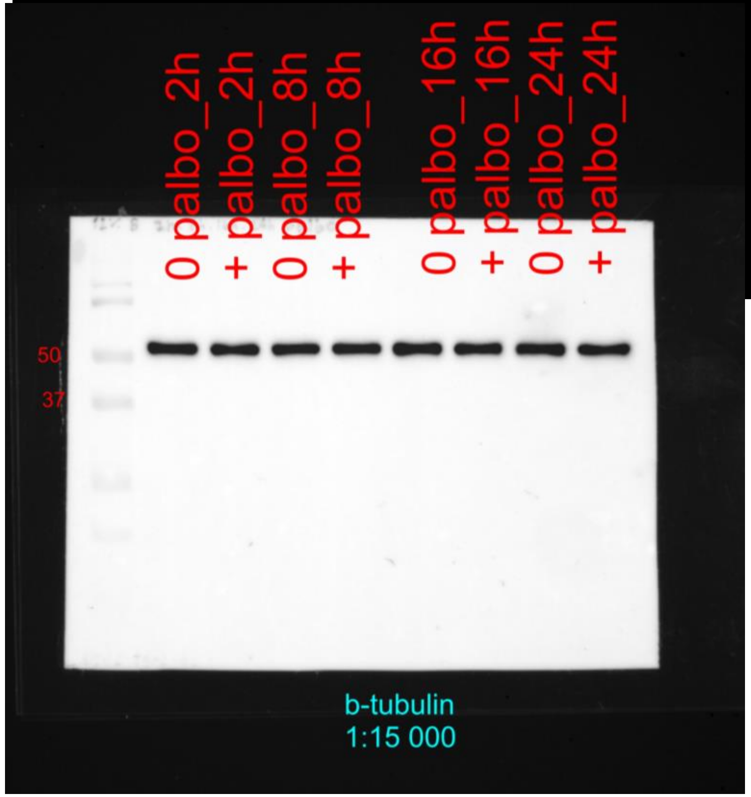
0 palbo_2h
+ palbo_2h
0 palbo_8h
+ palbo_8h
0 palbo_16h
+ palbo_16h
0 palbo_24h
+ palbo_24h
50
37
b-tubulin
1:15 000

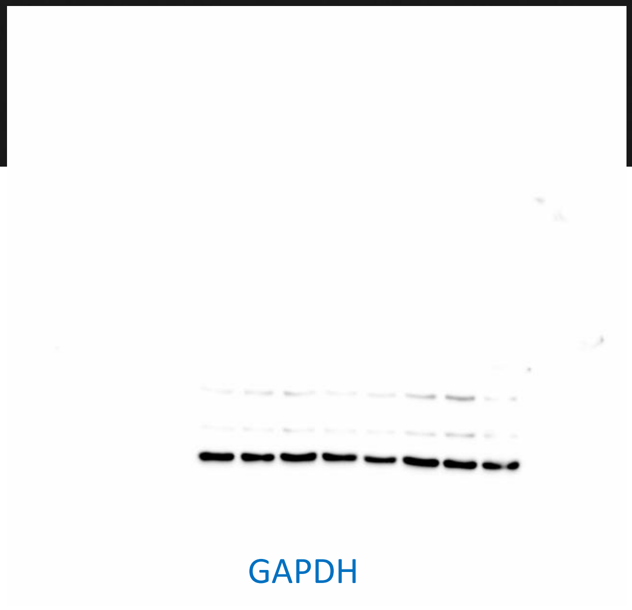
GAPDH

Suppl. Fig. 5a

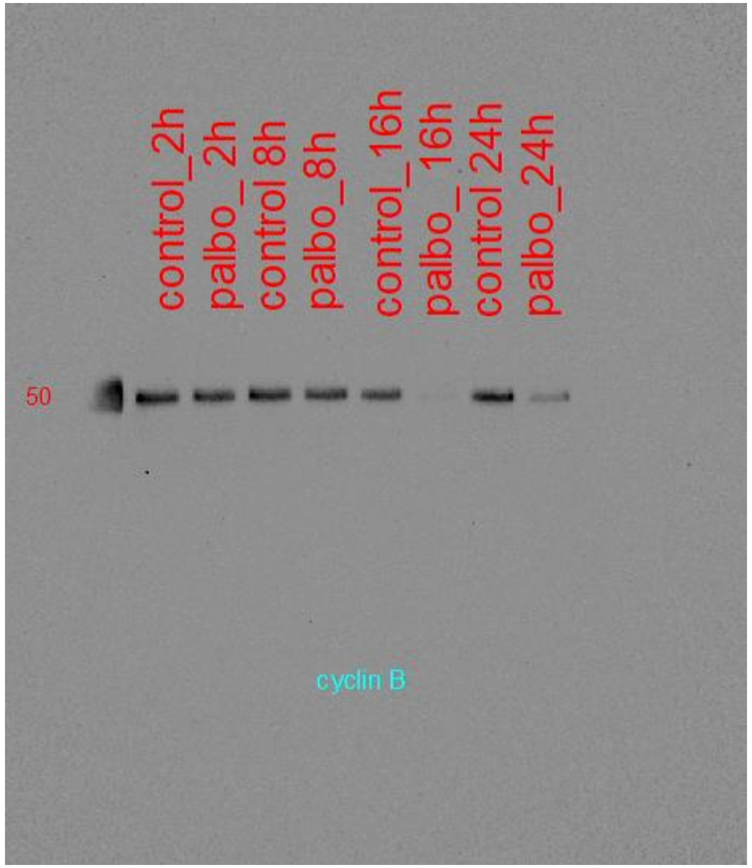
control_2h
palbo_ 2h
control 8h
palbo_8h
control_16h
palbo_ 16h
control 24h
palbo_24h
50
cyclin B

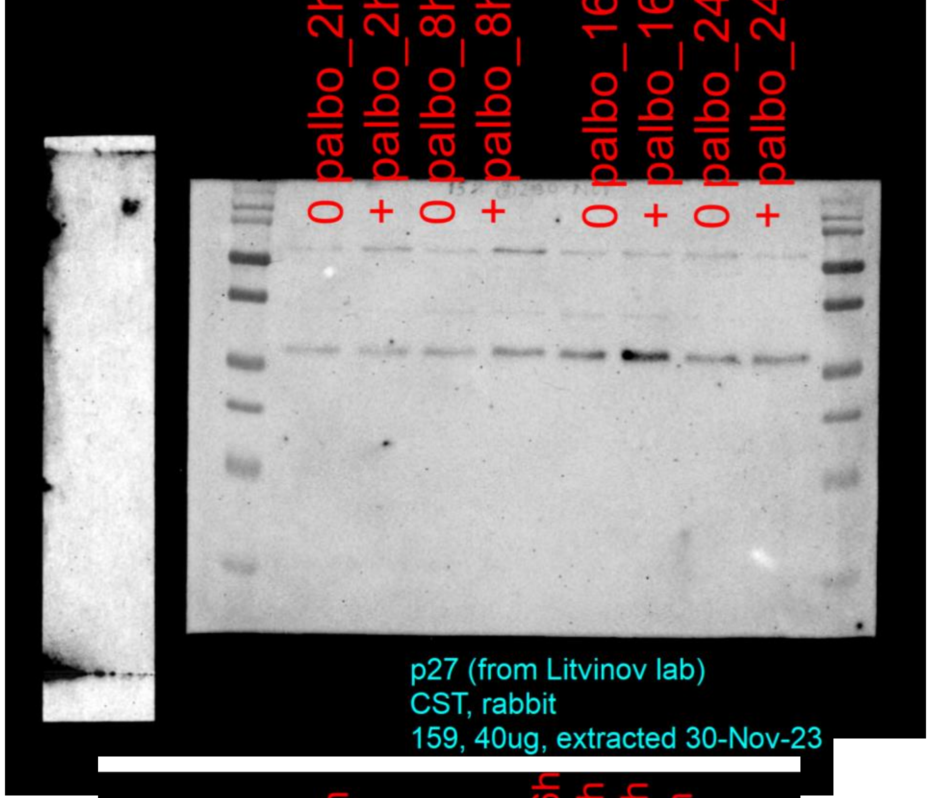
0
+
0
+
0
+
0
+
p27 (from Litvinov lab)
CST, rabbit
159, 40ug, extracted 30-Nov-23

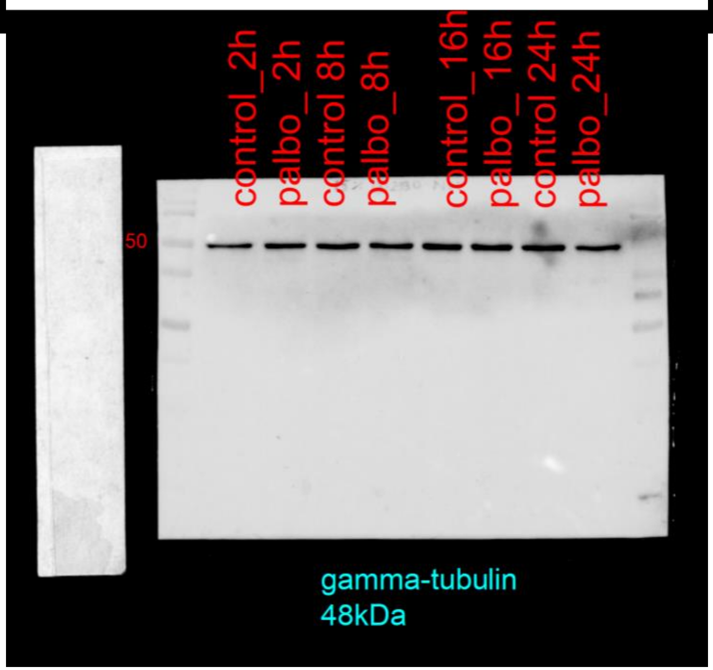
control_2h
palbo_ 2h
control 8h
palbo_8h
control_16h
palbo_ 16h
control 24h
palbo_24h
50
gamma-tubulin
48kDa

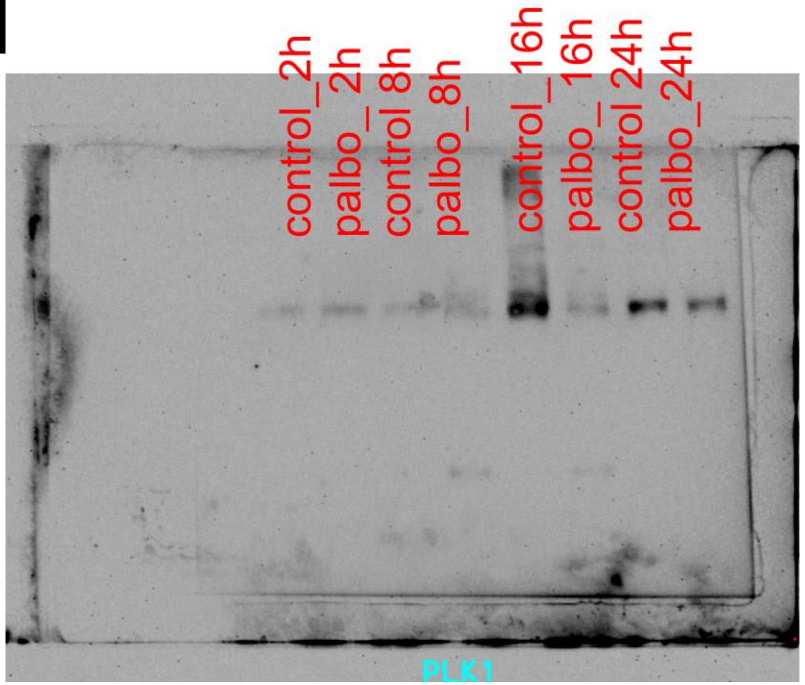
control_2h
palbo_ 2h
control 8h
palbo_8h
control_16h
palbo_ 16h
control 24h
palbo_24h
PLK1

Suppl. Fig. 5a

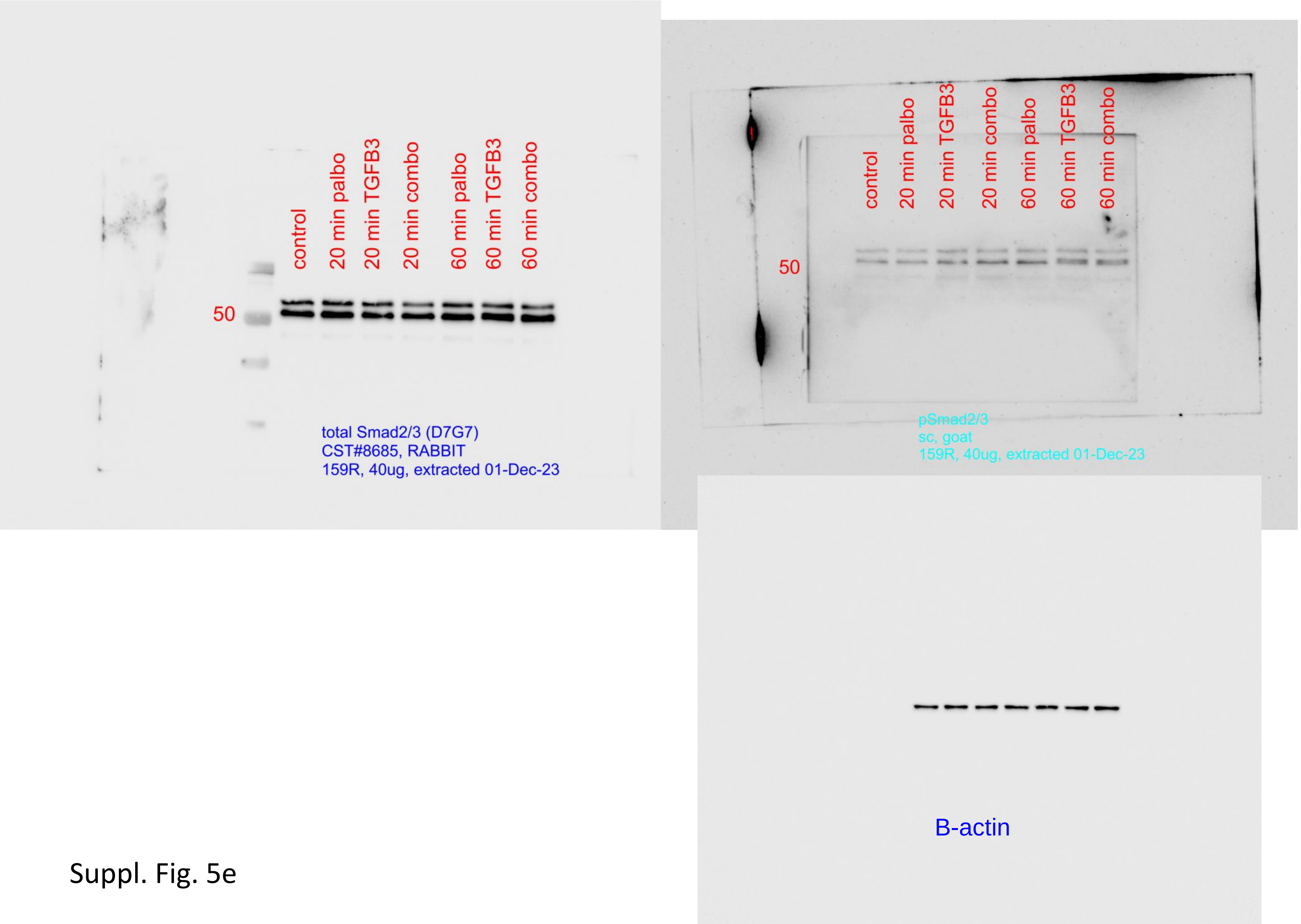
control
20 min palbo
20 min TGFB3
20 min combo
60 min palbo
60 min TGFB3
60 min combo
50
total Smad2/3 (D7G7)
CST#8685, RABBIT
159R, 40ug, extracted 01-Dec-23
control
20 min palbo
20 min TGFB3
20 min combo
60 min palbo
60 min TGFB3
60 min combo
50
pSmad2/3
sc, goat
159R, 40ug, extracted 01-Dec-23
B-actin

Suppl. Fig. 5e
